# The transcription factor FcMYB3 responds to ^60^Co γ-ray irradiation of axillary buds in *Ficus carica* L. by activating the expression of the NADPH oxidase, *FcRbohD*

**DOI:** 10.3389/fpls.2024.1476126

**Published:** 2024-11-26

**Authors:** Miaoyu Song, Ziyu Chen, Wupur Bahayiding, Jinping Li, Huiqin Ma, Ziran Wang

**Affiliations:** ^1^ College of Horticulture, Yunnan Agricultural University, Kunming, China; ^2^ College of Horticulture, China Agricultural University, Beijing, China; ^3^ Institute of Agricultural Sciences in Turpan, Xinjiang Academy of Agricultural Sciences, Turpan, China; ^4^ Fig and Walnut Research Institute of Weiyuan County, Weiyuan, China

**Keywords:** fig (*Ficus carica* L.), γ-Ray radiation, ROS, FcMYB3, FcRbohD

## Abstract

Plant irradiation has been used to induce genetic variation in crop germplasm. However, the underlying mechanisms of plant responses to ionizing radiation stress are still unclear. In plants, reactive oxygen species (ROS) are produced with abiotic stress. Respiratory burst oxidative homologs (Rboh) genes are important regulators of plant ROS stress responses, but little is known of their involvement in the response to ionizing radiation stress. In this study, young branches of *Ficus carica* L. were irradiated with ^60^Co γ-rays and axillary buds were collected after 3- 48 h after irradiation. The differentially expressed genes (DEGs; p< 0.05) detected included an early (6 h) and sustained increase in member of the MAPK signaling pathway. The activities of superoxide dismutase SOD, POD and CAT in fig axillary buds showed a trend of first decrease and then increase with time, while the contents of MDA and H2O2 maintained an overall upward trend. The analysis of differentially expressed genes (DEGs; p < 0.05) indicated an early (6 h) and sustained increase in member of the MAPK signaling pathway. DEGs for glutathione-s-transferase and genes involved in phenylpropanoid and flavonoid biosynthesis pathways were detected at all time points, indicating that γ-irradiation induced an increased capacity for in ROS-scavenging. Substantial changes in the expression of MYB, NAC and bHLH transcription factor family members were also seen to occur within 6 h after irradiation. Taking Rboh-derived ROS signaling pathway as the entry point, the MYB transcription factor, FcMYB3, was identified as an potential upstream regulator of *FcRbohD* in a yeast one hybrid assay and this interaction verified by LUC and EMSA experiments. The knock-down and overexpression of *FcMYB3* indicated that FcMYB3 is a positive regulator of ROS accumulation in response to γ-ray radiation stress responses in fig. Our results will provide a better understanding of the mechanisms of radiation tolerance in plant materials.

## 1 Background

Mutations in plants are defined as hereditary, molecular changes in the genome not caused by normal genetic recombination or segregation (Harten, 1998). Mutations provide a fundamental resource of genetic diversity and novel traits that cannot be introduced by the recombination of existing genomic sequences (Pathirana, 2021). Though mutations occur spontaneously, the high requirement for crop innovation has largely promoted the use of induced mutagenesis through various physical or chemical mutagens. More than 3200 mutagenized varieties of crops, ornamentals and trees, have been released for commercial use in different countries since the 1930s (International Atomic Energy Agency, https://www.iaea.org/topics/mutation-breeding).

Ionizing radiation (IR), including γ-ray, X-ray, ultra-violet (UV) light, and neutrons, are widely used as mutagenic agents and of these, γ-ray treatments have been the most successful for the development of new traits in crops (Nakagawara, 2009). Different species of plants vary greatly in their sensitivity to by ionizing radiation, with rates of mutagenesis between 1 × 10^−8^ and 1.2 × 10^−7^ base pair (bp) per 10 Gy (Caplin and Willey, 2018). The type of mutations observed in gamma-ray mutants have been listed as deletions, single base substitutions, inversions, transversions, translocations of chromosomes, and duplications in DNA (Shirasawa et al., 2016). Ionizing radiation acts directly and indirectly on DNA base-pairs and other cellular components. High-energy radiation causes direct ionization of DNA and leads to single- and double-strand breaks. The radiant energy also produces reactive oxygen species (ROS), such as superoxide anion (O_2_^–^), hydroxyl radicals (OH^*^), singlet oxygen (^1^O_2_), and hydrogen peroxide (H_2_O_2_) in the nuclei and other cellar compartments (Calucci et al., 2003; Lee et al., 2009; Wi et al., 2006). The ROS produced in response to ionizing radiation can cause changes in the deoxyribose ring, nucleotide structures, DNA-DNA and DNA-protein cross-links in the genome, while the increase in ROS in other cellular compartments can damage lipids, proteins and other biomacromolecules (Koyama et al., 1998). In order to maintain proper growth and development, plants have evolved complex survival mechanisms to resist stress, including physical adaptation and molecular and cellular changes. Through these mechanisms, plants can temporarily increase their tolerance to lethal radiation stress (Knight and Knight, 2001). In plants, ROS are known to serve as secondary messengers in cells, regulating many aspects of plant growth, development plant responses to environmental stress (Considine and Foyer, 2021). An important source of plant ROS signals are generated by NADPH oxidases (Rbohs) which catalyze the transfer of electrons from NADPH to O_2_, resulting in the production of superoxide anion (O_2_^•-^). Previous studies have shown that Rboh contain six transmembrane central regions, two heme groups, cytosolic FAD (flavin adenine dinucleotide) and NADPH (nicotinamide adenine dinucleotide phosphate) binding domains in the C-terminal (Sagi and Fluhr, 2006). The O_2_^•-^ generated by Rboh can be converted to H_2_O_2_ by peroxidases, thus affecting cell wall cross-linking by the same or other peroxidases (Passardi et al., 2004). In *Arabidopsis thaliana*, the *Rboh* gene family consists of 10 members and multiple Rboh genes have been identified in horticultural crops including rice, wheat, tomato, rapeseed, grape, citrus and tobacco (Zhang et al., 2022). Rboh is considered to be a central hub in the ROS signaling network and plays an important role in plant development (Liu et al., 2022; Hafsi et al., 2022; Lee et al., 2022). There is also substantial evidence for a pivotal role for Rboh genes in the responses to biotic and abiotic stresses at the transcriptional level (Zhang et al., 2015b; Hu et al., 2020). The RBOHD is a primary player in ROS production during innate immunity (Lee et al., 2020). Plants have developed a sophisticated immune system to defend against pathogens, consisting of two main layers: PAMP-triggered immunity (PTI) and effector-triggered immunity (ETI) (Boller and He, 2009). A key component in this system is RBOHD, which is essential for the ROS burst in response to bacterial PAMPs (Wang et al., 2020). Mutations in *Arabidopsis thaliana*’s AtRBOHD fully impair this oxidative response, underscoring RBOHD’s critical role in PTI defense (Su et al., 2023). However, there has been little research on the response of Rboh to radiation stress.

Rboh genes have been reported to be regulated by transcription factors including members of the MYB, NAC, AP2/ERF, WRKY, and bHLH families. The transmembrane motif 1-like 4 (NTL4) of the NAC family promotes ROS production by binding to the promoters of ROS biosynthetic genes (Lee et al., 2012). AtERF6 can bind to the *AtRbohD* promoter and positively regulates *AtRbohD* in the response to oxidative stress (Sewelam et al., 2013; Wang et al., 2013). NtbHLH123 can bind to the NtRbohE gene promoter to regulate the response of tobacco to salt stress mediated by ROS (Dan et al., 2021). Tomato SlWRKY81 acts as a negative regulator of drought tolerance by modulating H_2_O_2_-mediated stomatal closure through its effect on Rboh-derived H_2_O_2_ accumulation. Exogenous folic acid suppresses the expression of *VvRboh* gene associated with ROS production by downregulating the expression of VvWRKY31, thereby retarding the deterioration of grape quality induced by excessive ROS accumulation (Pei et al., 2023). The MYB family of transcription factors plays a key role in regulating ROS accumulation in plants by modulating the expression of H_2_O_2_-degrading enzymes (Su et al., 2023). For instance, BrMYB108 enhances ROS production by binding to the promoters of Rboh, contributing to broad-spectrum pathogen resistance (Su et al., 2023). In systemic tissues, AtMYB30, activated by the RBOHD-driven ROS wave, induces systemic acquired acclimation (SAA) and reduces ROS signaling, helping plants adapt to environmental stress (Fichman et al., 2021).

Fig (*Ficus carica* L) is an important and healthy component of the Mediterranean diet and one of the earliest fruit trees domesticated for cultivation (Flaishman et al., 2008). Figs, like most fruit tree crops are mainly vegetatively propagated through cuttings, which makes them ideal for controlled irradiation treatments to induce somatic mutation. In this study, axillary buds of young fig cuttings were used to investigate the enzymatic and transcriptional response to ionizing radiation in a time sequential manner. The results obtained provide new information on the specific biological pathways and genes involved in the response to ionizing radiation in this historical fruit tree. In addition, we used yeast one-hybrid (Y1H) screening, LUC and EMSA experimental methods to demonstrate that the MYB transcription factor, *FcMYB3*, can bind to the promoter region of the *FcRbohD* gene. *FcMYB3* plays an important role in the early stages of the radiation stress response by upregulating *FcRbohD* and enhancing ROS production. Functional tests indicated that *FcMYB3* regulates ROS homeostasis to enhance radiation tolerance. These findings suggest that the regulation of *FcMYB3* in response to gamma radiation stress is dependent on the Rboh-derived ROS signaling pathway.

## 2 Materials and methods

### 2.1 Plant materials and treatments

Young shoots were collected on June 7, 2020 from the common fig cv. Green Peel cultivated 2x3 m apart in a greenhouse at the Shang zhuang Experimental Station of China Agricultural University, Haidian District, Beijing. The original source of the fig materials used from Weihai Changshoukang Food Co., Ltd. in Shandong province. Experimental research on fig complies with relevant international and Chinese guidelines, and with Shandong province local legislation. The cuttings collected were from shoots ca. 50 cm in length with six to nine buds. Leaves were removed and the shoots covered with a wet cloth then subjected to treatment with ^60^Co-γ radiation. The radiation dosages used were 200, 100 or 50 Gy which, were delivered at 5 Gy/min from a distance of 37 cm. For each treatment, three replications were employed with 10 cuttings in each replicate. To determine the effect of irradiation on bud break, the irradiated shoots were cut into single node bud cuttings of about 10 cm, their source position on the shoot noted (apical, middle or basal) and inserted into holes of floated boards with the basal end in water. The bud break was counted after 11 days.

100 Gy was selected as the half-lethal dose for irradiation treatments. After irradiation, buds were collected from the shoots at 3, 6, 12, 24 and 48 h after shoot irradiation. For each sample, ≥45 buds were collected (ca. 3 g), Each of the 15 buds were a set of biological replicates, a total of three groups, rinsed with PBS/RNA-free water, blotted to dryness, frozen in liquid nitrogen and stored at -80 °C until further analysis.

### 2.2 Assays of antioxidant enzyme activities and malondialdehyde contents

Relevant enzyme activities and antioxidant indicators were determined according to the method of Nakano and Asada (Chikahiro et al., 1993). Of these, malondialdehyde (MDA) content was determined by the thiobarbituric acid method, superoxide dismutase (SOD) enzyme activity was determined by the nitrogen blue tetrazolium (NBT) photochemical reduction method, catalase (CAT) enzyme activity was determined based on the consumption of hydrogen peroxide (H_2_O_2_) per unit time, peroxidase (POD) enzyme activity was determined by the guaiacol method, and H_2_O_2_ content was determined was determined according to its precipitation of yellow complex with titanium chloride, dissolved by sulfuric acid and then determined by colorimetric method. Each sample assay was repeated with three technical replicates. Microsoft Excel 2016 software was used for data analysis and the results expressed as mean ± SD. The experimental data were subjected to ANOVA and compared with Duncan’s multiple polar difference test for the mean values.

### 2.3 RNA-seq, gene annotation and the detection of differentially expressed genes

RNA-seq and annotation were carried out as described previously. Briefly, the total RNA of fig bud or callus samples was extracted with a modified CTAB method (Wang et al., 2021) and tested for integrity by 1% agarose gel electrophoresis. The total RNA concentration and purity were verified by NanoDrop 2000 (NanoDrop Technologies, Wilmington, DE, USA) and Agilent 2100 (Agilent Technologies, Palo Alto, CA, USA), respectively. cDNA was synthesized using a cDNA Synthesis Kit (TaKaRa, Dalian, China) and the sequencing adapter was linked to both ends. Library construction was as described previously (Wang et al., 2018). High-throughput sequencing was carried out using an Illumina HiSeq 4000 platform (Illumina, Shanghai).

The fig bud transcripts were annotated against the SwissProt (SwissProt) and Nonredundant (Nr) protein databases. For gene-expression analysis, reads mapped to each gene were counted using HTSeq v0.5.4p3 and then normalized to FPKM (fragments per kilobase of transcript per million mapped reads (Langmead and Salzberg, 2012). Differentially expressed genes (DEGs) between experimental conditions were selected based on log2 fold changes ≥ 1 and false detection rates ≤ 0. 05. All genes were annotated with terms for Gene Ontology (GO), Cluster of Orthologous Groups (COG), Protein families data-base (Pfam) and Kyoto Encyclopedia of Genes and Genomes (KEGG) The whole set of annotated genes can be found in the National Center for Biotechnology Information (NCBI) SRA database with the accession number (PRJNA1144934).

### 2.4 Bioinformatics analysis of the *FcRbohD* gene and protein sequence

The physical and chemical properties of the FcRbohD protein were analyzed using the online protparam package (https://web.expasy.org/protparam/). SignalP -4.1 was used online (https://services.healthtech.dtu.dk/service.php?SignalP-4.1) to predict the presence and position of the signal peptide. The subcellular localization was predicted using Wolf PSORT (https://wolfpsort.hgc.jp/). The prediction of transmembrane domains utilized tmhmm 2.0 at (https://services.healthtech.dtu.dk/service.php?TMHMM-2.0) and Netphos3.1 was used to predict phosphorylation sites (https://services.healthtech.dtu.dk/service.php?NetPhos-3.1) The secondary and tertiary structures of FcRbohD protein were predicted by the online software SOPM (https://npsa-prabi.ibcp.fr/cgi-bin/npsa_automat.pl?page=npsa_sopma.html#opennewwindow) and SWISS-MODEL(https://swissmodel.expasy.org/), respectively. The 1307 bp sequence upstream of the transcription starting point of FcRbohD was selected as the promoter region for analyzing cis acting elements. All cis-acting elements in this region were identified using the online resource, PlantCare (http://bioinformatics.psb.ugent.be/webtools/plantcare/html/) and the important response elements were screened out Excel. The selected cis-elements and then mapped by tbtools.

### 2.5 Yeast one-hybrid assay

The primers used to obtain cDNA sequence of the *FcMYB3* CDS and the promoter region of *FcRbohD* were designed with Primer Premier 5.0 (Premier Biosof) with reference to the fig genome (Mori et al., 2017) and consisted of *Fcmyb3*-F-ATGTAGGCCTTCTTCTCTCTC, *Fcmyb3*-R GGCCTTAATCGTCCCTTTCC, *FcRbohD* used the forward-F-ATGAAAAGACACGCTTACAT and *FcRbohD*-R-CAAGTGGTGGAGTTGAATCA. PCR was carried out with a Q5^®^ High-Fidelity DNA Polymerase (New England Biolabs, Ipswich, MA, USA) as per the manufacturer’s recommendations.

The yeast one-hybrid system (Y1H Gold) was used to screen for potential interactions the relationship between *FcMYB3* and the *FcRbohD* promoter. For the effector construct, the open reading frame of *FcMYB3* were cloned into the SmaI and SacI sites of the pGAD-T7 vector. The *FcRbohD* promoter sequence was inserted upstream of the AbAr reporter gene of the pABAi vector. The following decoy reporter strains were also produced: AD-empty/pABAi-pFcRbohD -pro and AD-p53/pp53. The transformation, growth and yeast colony selections were performed as recommended by the manufacturer.

### 2.6 Dual-luciferase assay

The full-length CDS sequence of *FcMYB3* and the promoter sequence of *FcRbohD* were inserted into the pCambia 1300-35S and the pGreenII-0800-LUC vectors, respectively, and then both transformed into competent cells of *Agrobacterium* GV3101. combinations of blank vectors were used as controls. Colonies transformed with both vectors were selected for by growth on solid Luria broth (LB) media containing kanamycin, then single colonies were cultured in liquid LB medium at 28°C with shaking until an OD_600_ of ca. 0.8. After pelleting by centrifugation at 4,500 g for 5 min, the *Agrobacterium* cells were resuspended in infestation solution (MgCl_2_) and 2 mL injected it into the abaxial surface of tobacco leaves. The leaves were incubated for 2 d under darkness before imaging of fluorescence at the injection site, using a live fluorescence imager (NightSHADE L985, Berthold, Germany).

### 2.7 Electrophoretic mobility shift assay

The CDS of FcMYB3 was inserted into pGEX-4 T-1 in frame with the N-terminal GST tag, and then the construct transformed into strain BL21 for bacterial expression. The FcMYB3-GST fusion protein was expressed under 0.3 mM IPTG at 37°C for 8 h and purified using a Pierce™ GST Spin Purification Kit (Thermo Fisher Scientific, Beijing). The *FcRbohD* promoter fragment containing the sequence TAACTG was labeled with biotin A LightShift™ Chemiluminescent EMSA Kit (Thermo Fisher Scientific, Pierce™ Biotin 3’ End DNA Labeling Kit) was used to detect mobility shifts following the manufacturer’s instructions. The unlabeled promotor fragment was used to verify bands. The signals were captured using the ChemiDoc Imaging System (Bio-Rad, XXX). The primers are listed in **Supplementary Table S1**.

### 2.8 VIGS transient silencing and overexpression of *FcMYB3*

VIGS in plants outperforms earlier gene silencing methods, offering long-lasting and transmissible post-transcriptional and transcriptional silencing (Beyaz, 2023). The CDS of *FcMYB3* obtained above was inserted into the virus silencing vector, pTRV2. the pTRV2- FcMYB3 constructs were then ligated into the pCambia 1300-35S vector and transferred into Agrobacterium GV3101 cells as described above. Agrobacterium GV3101 cells harboring pTRV1 constructs were obtained from our laboratory. For the overexpression of *FcMYB3*, Agrobacterium GV3101 cells transformed with pCambia 1300-35S- *FcMYB3* was utilized as described for the LUC assay above.

*F. carica* callus was derived from fig leaves (MS medium containing 30g/L fructose, 2mg/L TDZ, 2mg/L 6-BA, 0.05mg/L NAA, and 7.5mg/L Agar with VC at pH range of 5.84-5.87) and incubated in the dark at room temperature (25°C) for 20 days prior to subculture. Subsequently, the callus was meticulously selected using tweezers and transferred to a new tissue culture bottle containing 40ml of sterile water, with one-third volume of callus, heavy suspension bacteria, and 10-20ul AS. The mixture was then gently agitated at room temperature (120-150 rpm) for 30 minutes. After filtration through 2-3 layers of gauze and discarding the filtrate, the callus was allowed to rest for 5 minutes before being transferred to a secondary plate medium without spreading out but forming clumps. It was then cultured in the dark for 8-12 hours.

The various Agrobacterium GV3101 lines described above were then used to transform *F. carica* callus for the knock-down (VIGs) and overexpression of *FcMYB3*. The positive callus obtained by liquid nitrogen treatment was used. The positive Calli were identified after dark culture for 3 days.

### 2.9 Expression analysis by RT-qPCR

RNA extraction, quality control and cDNA synthesis for RT-qPCR were carried out as described for RNA-Seq. 20 DEGs identified from the RNA-Seq analyses were randomly selected for validation. The primers used for specific genes were designed using Primer Premier 5 software (Premier Biosof, Kunming) and are given in **Supplementary Table S1**. qRT-PCR reactions utilized the Ultra SYBR Mix kit (TaKaRa, Dalian, China) with 10 μL UltraSYBR Premix System II, 1 μL of each primer (10 μM), 2 μL cDNA and 6 μL ddH2O. Three biological replicates were prepared for each sample. The amplification program was 95 °C for 10 min, followed by 40 cycles of 95 °C for 15 s and 58 °C for 1 min using a 7500 Fast Real-Time Detection System (Applied Biosystems, Kunming, China). For data analysis, relative quantification analysis was performed using the comparative CT (2^-ΔΔCT^) method with β-actin as the internal reference gene. The significance of differences between two groups was assessed by two-tailed Student’s t-tests, and for multiple groups were calculated using one-way analysis of variance (ANOVA) followed by Duncan’s test. Analyses were performed using SPSS Version 16.0, with significance set at *P* < 0.05.

## 3 Results

### 3.1 The effect of 60Co-γ radiation of Green Peel Fig shoots on bud break

The proportion of buds breaking at 11 d after ^60^Co-γ irradiation showed a significant downward trend with increasing radiation doses (**Table 1**). The position of the buds on the young shoot (apical, middle and basal nodes) was also seen to have an effect on their radiation tolerance. **Table 1** shows that at 200 Gy, the buds presented the lowest bud break (7.07%) at the shoot base. At 100 Gy, apical buds had the lowest survival rate, while those of mid and basal buds were relatively higher (56.2-57.6%). At the treatment dosage of 50 and 100 Gy, buds on nodes from the middle section had a slightly higher bud breaking rate than those from the upper part. In general, the bud break of each part decreased over time, and the proportion of bud breaks from mid and apical shoot sections were higher than from the basal section. In contrast, the proportion of buds breaks from control shoot nodes increased gradually during the monitored period (11 days), with no significant differences observed between bud locations. Based on these results, 100 Gy was selected as the radiation treatment for use in further analyses.

**Table 1:** Bud breaking rate of Green Peel fig young shoot cuttings at 11 days after ^60^Co-γ radiation.

Dose (Gy)	Shoot cutting position on the branch	Bud breaking rate (%)
0	Up	94.20 ± 1.45 d
	Middle	95.83 ± 2.41 d
	Base	85.33 ± 2.67 c
50	Up	74.07 ± 2.45 c
	Middle	87.88 ± 4.63 c
	Base	73.12 ± 3.88 b
100	Up	40.86 ± 4.96 b
	Middle	57.58 ± 1.75 b
	Base	56.25 ± 1.80 a
200	Up	10.00 ± 1.93 a
	Middle	22.22 ± 1.60 a
	Base	7.07 ± 1.01 a

Different letters represent significant difference (P ≤ 0.05).

### 3.2 Transcriptome analysis

After the removal of linker and low-quality sequences, each of the RNA-Seq libraries prepared from irradiated buds after 0 -48 h produced between 7.77-8.98 Gb of clean data (**Supplementary Table S2**). A total of 106,376 unigene sequences were obtained after redundant sequences were removed.

**Figure 1:**
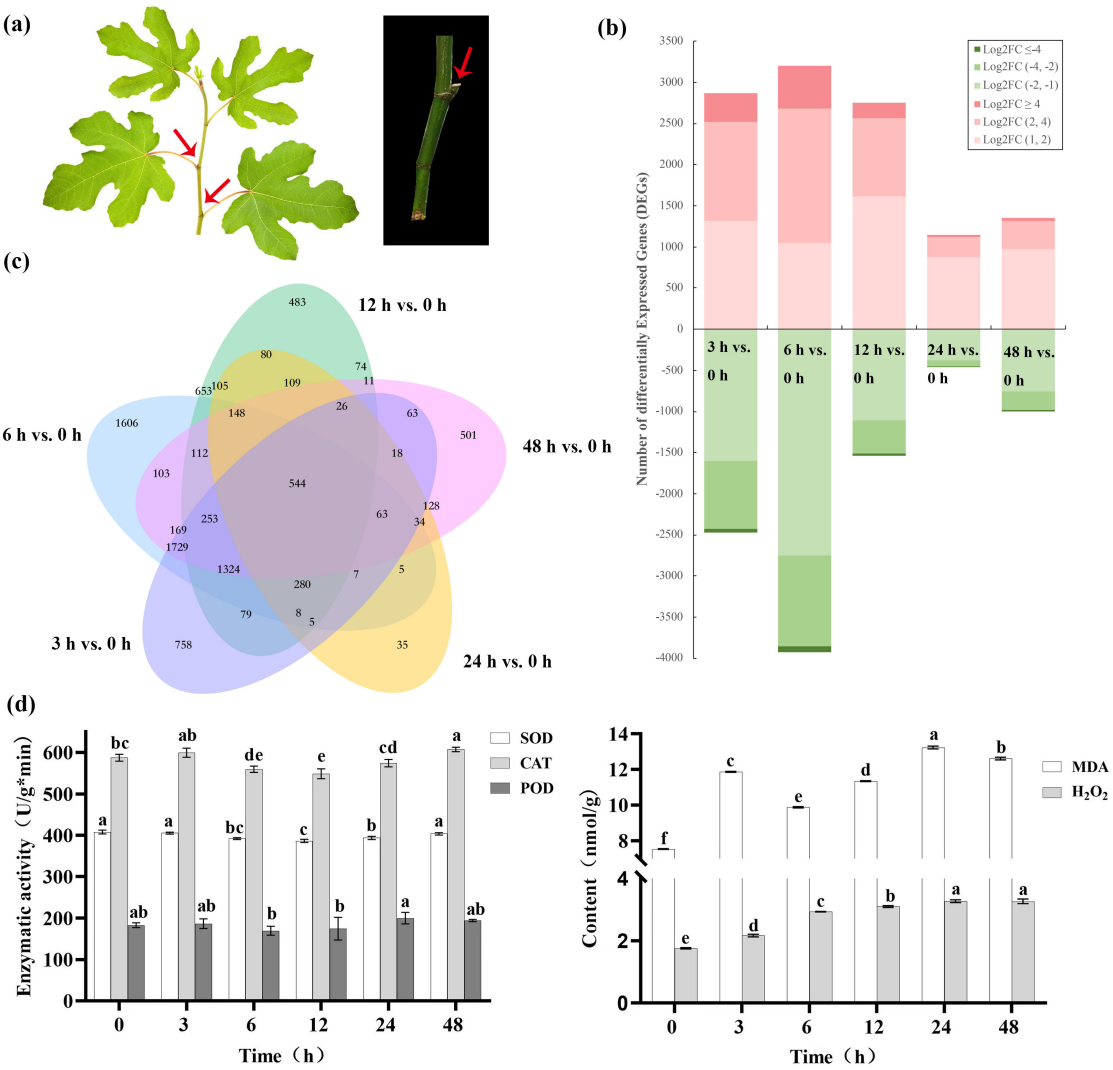
Gene expression character of Green Peel fig young shoot axillary buds after 100 Gy γ-radiation. **(A)** Green Peel fig young shoot and axillary buds. **(B)** Transcript abundance regulation. FC, fold change. **(C)** Corresponding Venn diagrams of DEGs at 3, 6, 12, 24 and 48 h after radiation. **(D)** Effect of radiation on axillary bud enzyme activity. Different letters represent significant difference (P ≤ 0.05).

A typical fig shoot after cutting is displayed in **Figure 1A**, we cut into fig shoots around 50 cm in length which was with six to nine buds on each shoot. An analysis of the RNA-Seq data for DEGs, indicated that relative to the control (0 h after irradiation), a larger number of DEGs could be detected during the first 12 h after irradiation (4289-7135) than after 24–48 h (1595-2356). The number of upregulated DEGs, 2869, 3205, 2752, 1146 and 1352 genes were upregulated, and 2468, 3930, 1537, 449 and 1004 genes downregulated in the sequential comparisons listed above, respectively. Furthermore, the highest changes in DEGs (FCs >= 4) more frequently occurred in upregulated genes (**Figure 1B**), which supports an overall positive effect of radiation treatments on DEG expression. An analysis of the DEGs showed that, 544 DEGs were common to all samples 3–48 h after irradiation (**Figure 1C**). GO analysis assigned 24983, 31125 and 29764 unigenes to biological processes, cell component localization and molecular functional class, respectively (**Supplementary Figure S1A**).

An analysis of DEGs for KEGG pathway enrichment demonstrated some interesting changes in response to irradiation (**Supplementary Figure S1B**). Changes in the pathway for error-free homologous recombination was the third most significant after 3 h, but insignificant at later time points (**Table 2**). Pathways of DNA replication and base excision repair were also found to be significantly enriched at 3 h, indicating an early response to DNA damage (**Table 2**). Significant changes in the MAPK signaling pathway was first detected at 3h (9^th^ most significant response) but became more significant at later time points (first or second most significant response), indicating an important role for kinase cascades in the fig response to irradiation. Pathways for stilbenoid, diarylheptanoid, gingerol, phenylpropanoid and flavonoid biosynthesis were significantly altered in all irradiated samples, highlighting the specific major pathways for ROS scavenging in the fig bud response to acute irradiation (**Table 2**).

**Table 2:** Significant KEGG pathways (corrected *P* -value ≤ 0.01) of differentially expressed genes (DEGs) of Green Peel fig axillary buds after ^60^Co γ-ray radiation mutagenesis.

No.	Pathway	DEGs with pathway annotation**	Corrected *P*-value
	3 h vs. 0 h		
1	Plant hormone signal transduction	54	1.03E-05
2	Phenylpropanoid biosynthesis	50	1.2E-05
3	Homologous recombination	31	0.000752
4	Diterpenoid biosynthesis	10	0.001059
5	Stilbenoid, diarylheptanoid and gingerol biosynthesis	17	0.00111
6	DNA replication	28	0.001281
7	Flavonoid biosynthesis	19	0.002219
8	Starch and sucrose metabolism	43	0.002599
9	MAPK signaling pathway - plant	33	0.002741
10	Base excision repair	18	0.00327
11	alpha-Linolenic acid metabolism	17	0.004143
	6 h vs. 0 h		
1	Phenylpropanoid biosynthesis	59	2.53E-05
2	MAPK signaling pathway - plant	44	0.000473
3	Plant-pathogen interaction	72	0.000554
4	Starch and sucrose metabolism	56	0.000642
5	Plant hormone signal transduction	57	0.001603
6	Stilbenoid, diarylheptanoid and gingerol biosynthesis	18	0.008432
7	Flavonoid biosynthesis	21	0.009945
	12 h vs. 0 h		
1	MAPK signaling pathway - plant	40	1.1E-08
2	Phenylpropanoid biosynthesis	47	1.56E-08
3	alpha-Linolenic acid metabolism	22	1.18E-07
4	Plant-pathogen interaction	55	1.42E-06
5	Starch and sucrose metabolism	36	0.002026
6	Stilbenoid, diarylheptanoid and gingerol biosynthesis	14	0.002095
	24 h vs. 0 h		
1	MAPK signaling pathway - plant	26	7.6E-11
2	Flavonoid biosynthesis	13	9.53E-07
3	Linoleic acid metabolism	8	8.08E-06
4	Phenylpropanoid biosynthesis	22	7.83E-06
5	alpha-Linolenic acid metabolism	10	8.05E-05
	48 h vs. 0 h		
1	MAPK signaling pathway - plant	33	1.13E-08
2	Plant hormone signal transduction	41	2.19E-08
3	Flavonoid biosynthesis	18	2.19E-06
4	Phenylpropanoid biosynthesis	29	0.000338
5	Carotenoid biosynthesis	11	0.000377

**FDR < 0.05 and absolute value of Log_2_FC ≥ 2 (2-fold) as the threshold.

To validate the key results of the RNA-Seq, we randomly selected 20 genes (RPA:3, RFC:3, POLD:2, DNA repair:2, Peroxidase:4, GST:4, MYB:2) and analyzed their expression levels at 0 h, 3 h, 6 h, 12 h, 24 h and 48 h post-irradiation by RT-qPCR. **Supplementary Figure S2** demonstrates a strong correlation between the expression levels of these genes and their RNA-Seq data, indicating the reliability of the RNA-Seq results.

### 3.3 Effect of radiation on axillary bud enzyme activity

Following irradiation, SOD activity of fig axillary buds showed significant decreases (*P* < 0.05) at 6–24 h 3.50–5.28%, after which values were insignificantly different from control levels. The change pattern of POD activity was consistent with that of SOD, decreasing by 4.67–7.26% between 12–24 h, but was not significant (*P* ≥ 0.05). CAT activity also showed a similar, but insignificant decreases between 6–24 h, but significantly increased after 48 h. 48 h treatment showed a significant increase of 1.03-fold after 48 h. MDA content showed a complex trend of increasing, then decreasing and then increasing. There were two peaks during this period, the MDA content 12 h after irradiation was 11.86 nmol/g, 57.50% higher than that of the control. After 24 h, the MDA content reached 13.22 nmol/g, 33.94% and 16.58% higher than that after 6 h and 12 h, respectively. The H_2_O_2_ content showed an increasing trend with increasing time after irradiation, reaching 3.27 nmol/g in after 48 h and achieving significant differences to the control after 6 (**Figure 1D**).

### 3.4 DEGs in ROS signaling pathway and *RbohD* gene screening

**Figure 2:**
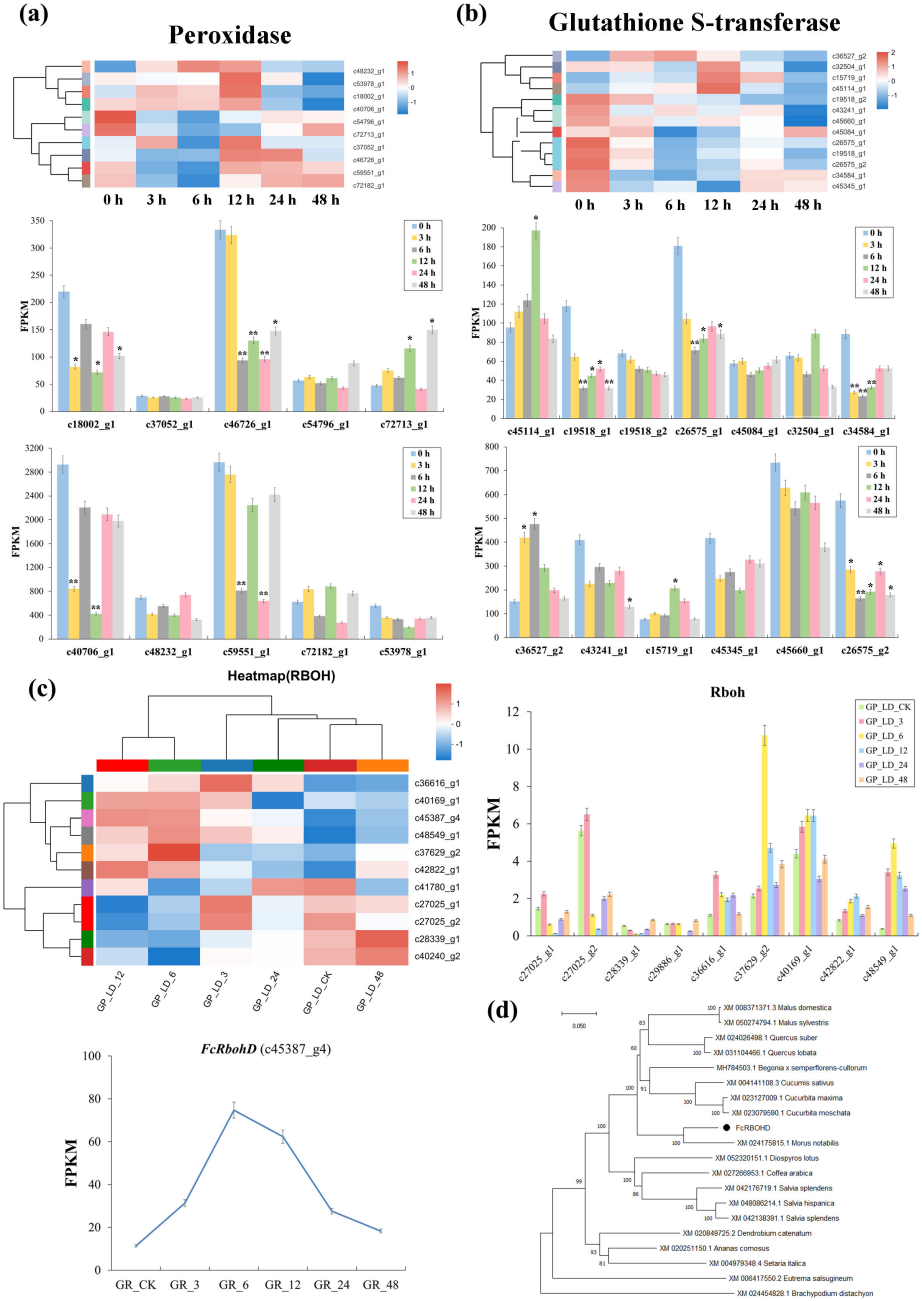
DEGs in ROS signaling pathway and *FcRbohD* gene screening of Green Peel fig young shoot axillary buds after 100 Gy γ-radiation. **(A)** Expression change of peroxidase genes coding direct ROS scavengers. **(B)** Expression change of Glutathione-S-transferase genes coding direct ROS scavengers. **(C)** Differential expression analysis of RbohDs gene and amino acid sequences analysis of *FcRbohD* gene. **(D)** The phylogenetic tree analysis of *FcRbohD* gene. *, significance level at 0.05; **, significance level at 0.01.

At the transcriptional level, the response to irradiation was seen to involve significant changes in peroxidases, glutathione-S-transferases and Rbohs, suggesting a significant contribution from the oxidative stress response. The number of three peroxidase genes detected showed up-regulation at 3 and 6 h after irradiation, but were reduced at following time points. However, seven peroxidases showed a generally downward trend, while three others displayed increases until 48 h. The remaining two peroxidase DEs showed differing trends. c59551_g1 showed a significant downregulation of 1.56- and 1.68-fold, at 3 and 6 h, respectively while c46726_g1 was significantly up-regulated at 12 and 24 h with fold changes of 2.57- and 2.32, respectively (**Figure 2A**).

Twenty-four glutathione-S-transferases GSTs were found to show differential expression. Ten of these (FPKM ≥ 20) demonstrated similar changes in expression to those observed in the majority of peroxidase DEGs, with upregulation at 3 and 6 h after irradiation, followed by a gradual decrease thereafter. However, the remaining GST DEGs detected showed significantly differing profiles (**Figure 2B**).

A total of 11 Rboh genes were observed to differentially expressed after radiation treatment. However, the c45387_g4 showed both FPKM values ≥ 20 and significantly elevated expression (**Figure 2C**) and was therefore selected for further analysis. The amino acid sequence of the c45387_g4 gene and orthologous sequences were used to construct phylogenetic tree (**Figure 2D**), where c45387_g4 gene can be seen to closely clustered 99.69% RbohD of Sankoh (99.69% sequence similarity), and was therefore named FcRbohD.

### 3.5 Bioinformatics and promoter analysis of *FcRbohD*

**Figure 3:**
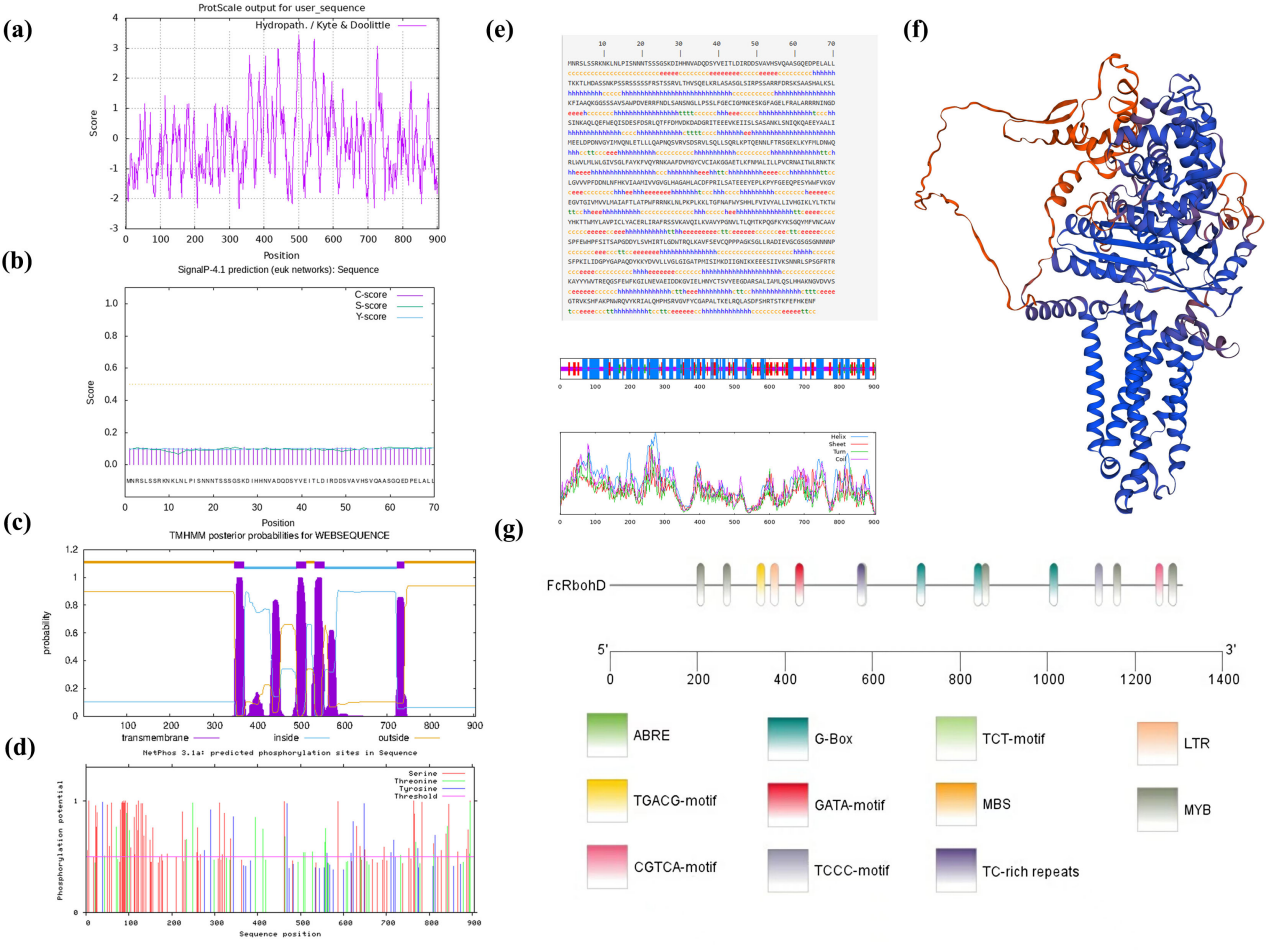
Bioinformatics and promoter analysis of FcRbohD. **(A)** Hydrophilicity prediction of the FcRbohD protein. **(B)** Signal peptide analysis in the FcRbohD protein. **(C)** Transmembrane domain prediction in the FcRbohD protein. **(D)** Phosphorylation site prediction in the FcRbohD protein. **(E)** Secondary domain prediction for the FcRbohD protein. **(F)** Tertiary domain prediction of the FcRbohD protein. **(G)** Analysis of cis-acting elements in the promoter region of FcRbohD.

The predicted score of amino acid at position 500 in the peptide chain was the highest (3.444), and the predicted score of amino acid at positions 327 and 328 in the peptide chain was the lowest (-2.322). Therefore, FcRbohD was an unstable hydrophilic protein (**Figure 3A**). FcRbohD is predicted to be a 905 amino acid (101.4 kD), and basic (pI of 9.2) and non-secreted protein (**Figure 3B**). Like other Rbohs, FcRbohD is predicted to be an integral plasma membrane-localized protein with four transmembrane regions (**Figure 3C**). The protein instability coefficient of FcRbohD was 46.87, the protein fat coefficient was 88.14, and the average hydrophilicity coefficient was -0.205.

FcRbohD is predicted to contain 103 phosphorylation sites, which are mostly serine (68), followed by threonine (21) and tyrosine (14) (**Figure 3D**). The secondary structure of FcRbohD was predicted to consist of 45.30% α-helix, 33.59% random coil, 16.13% extended strand and 4.97% β-turn (**Figure 3E**). The tertiary structure of FcRbohD protein was predicted and is shown in **Figure 3F**.

An analysis of the *FcRbohD* promoter (-1305 bp) for *cis*-acting elements indicated the presence of response elements for low temperature (LTR), drought (MBS), light (G-box, GATA, TCCC and TCT motifs), and hormones, including gibberellin (ABRE) and methyl jasmonate (TGACC and CGTCA motifs). Multiple MYB binding sites were also detected. This indicated that the expression of *FcRbohD* gene is likely to be affected by stress, phytohormones, light and other environmental factors (**Figure 3G**).

### 3.6 Transcription factors

After ^60^Co-γ radiation, a large number of transcription factors (TFs) were seen to be differentially regulated. These mainly consisted of members of the MYB, WRKY, AP2/ERF and bHLH families (**Table 3**). The MYB family of TFs was seen to contain the largest number of DEGs. It is well known that MYBs are involved in the regulation of phenylpropanoid and flavonoid biosynthesis, and MYBs could play important roles in plant stress resistance and cellular senescence. c44569_g1, annotated as MYB3, showed the highest FPKM value (3098.87) at 3 h, corresponding to a significant upregulation of 9.94-fold. Conversely, MYB34-c36961_g3 showed significant downregulations ranging from FCs of 2–5.2 from 3–48 h after irradiation. Similarly, c38505_g2, annotated as c-MYB-3R-1, showed a trend of downregulation of -1.57, -2.00 and -1.67 fold at 3, 6 and 12h, respectively.

**Table 3:** Expression profiles of same and differentially expressed genes (DEGs) encoding transcription factors (TFs) in Green peel fig dormant branch by ^60^Co γ-ray radiation mutagenesis.

TFs name	No. of TFs	Upregulate	Downregulate	No. of TFs	Upregulate	Downregulate	No. of TFs	Upregulate	Downregulate	No. of TFs	Upregulate	Downregulate	No. of TFs	Upregulate	Downregulate	Biological functions
	3 h vs. 0 h			6 h vs. 0 h			12 h vs. 0 h			24 h vs. 0 h			48 h vs. 0 h			
MYB	26	13	13	29	15	14	26	15	11	12	8	4	12	6	6	Cell development, anthocyanin pathway
WRKY	25	22	3	31	28	3	26	26	0	22	22	0	11	10	1	Defense responses
AP2/ERF	24	13	11	31	20	11	26	18	8	10	10	0	13	9	4	Plant development, stress response
bHLH	15	4	11	20	8	12	12	9	3	4	4	0	10	7	3	Plant development, substance metabolism
HSF	5	4	1	8	7	1	5	4	1	4	2	2	5	2	3	Plant development, stress response
bZIP	3	2	1	5	5	0	7	5	2	2	2	0	1	1	0	Photomorphogenic, fruit ripening
E2F	2	2	0	3	3	0	3	3	0	1	1	0	−	−	−	Cell regulation, apoptosis
OFP	2	0	2	3	0	3	1	0	1	−	−	−	2	1	1	Fruit development, DNA repair
NAC	−	−	−	−	−−	−	1	1	0	−	−	−	−	−	−	Fruit development, plant stress response
Others	56	21	35	69	28	41	29	19	10	13	10	3	19	13	6	
Total	158	81	77	199	114	85	135	99	36	68	59	9	109	68	41	

*P-adjust value ≤ 0.01 and |log_2_ FC| ≥ 2 as the threshold.

Eight heat shock factors (HSFs) showed altered expression at 6 h after radiation treatment, of which seven were upregulated and one downregulated. c40712_g2 and c43108_g1 HSFs were significantly up-regulated by 6.23- and 5.51-fold at 6 h.

An Ovate family transcription factor, c79834_g1, was down-regulated at 3, 6 and 12 h while the FPKM value was increased from 11.695 to 31.325 at 48 h. Three E2Fs (c30313_g1; c46505_g1; c39980_g1) also showed upregulation at 3 h, 6 h, 12 h and 24 h. E2F-c30313_g1 showed positive fold-changes of 3.75, 3.39 and 2 at 3, 6 and 12 h, respectively (**Table 3**).

### 3.7 FcMYB3 regulates ROS scavenging in the early stages of γ-ray radiation stress

**Figure 4:**
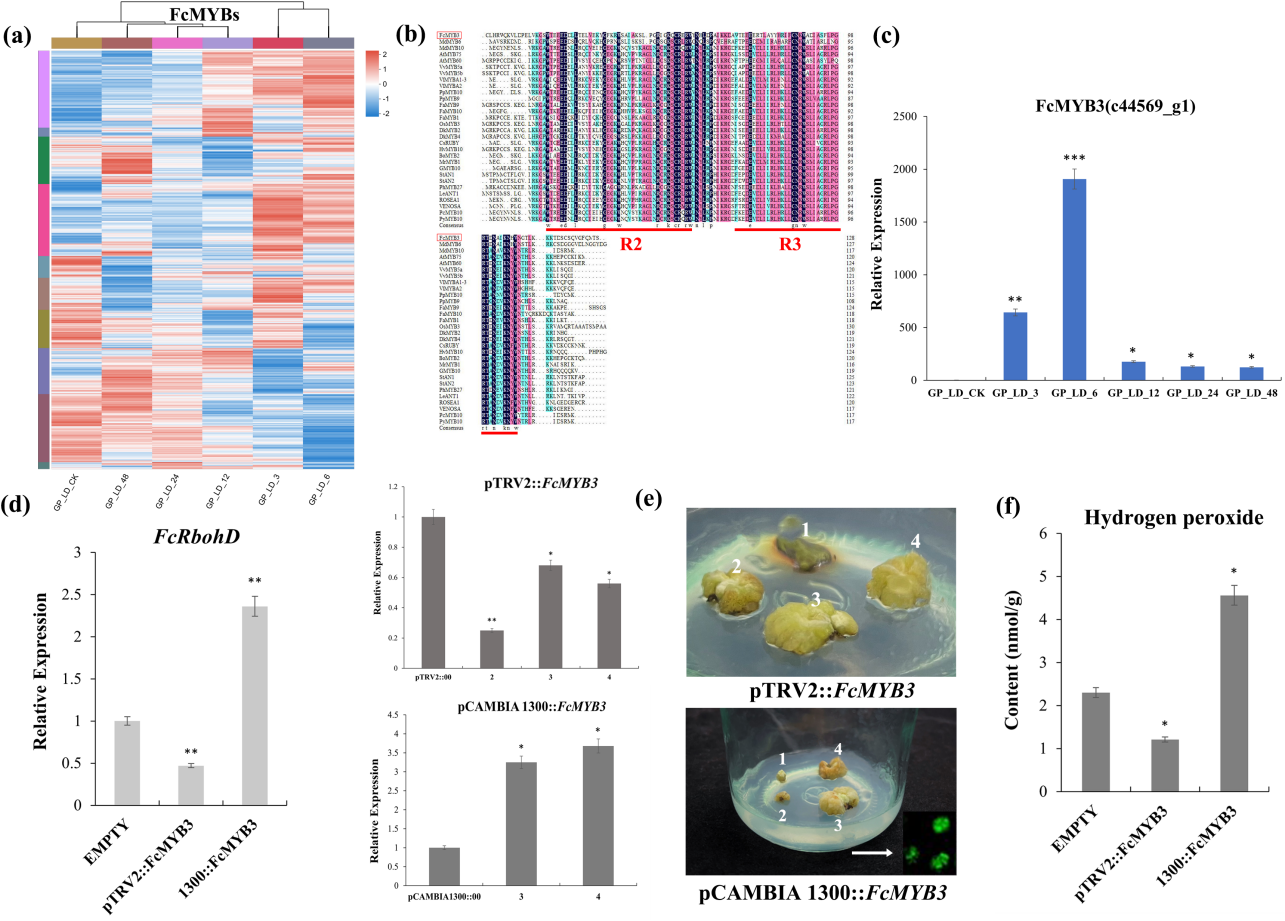
Screening and functional validation of the FcMYB3 gene. **(A)** The significantly differentially expressed MYB DEGs. **(B)** FcMYB3 gene expression trend. **(C)** The multiple sequence comparisons of FcMYB3 gene. **(D)** RT-qPCR analyses of transient knock-down (VIGS) and over-expressing (OE) *F. carica* callus of FcMYB3 gene. **(E)** The transiently transformed callus in the dark. **(F)** OE callus content of increased H_2_O_2_. The statistical significance was determined using Student’s t-test (*P < 0.05; **P < 0.01; ***P < 0.001).

A total of 43 significantly differentially expressed MYB DEGs displayed differential expression at 48 h after radiation treatment with FPKMs > 20 (**Figure 4A**). Of these, c44569_g1 (FcMYB3) gene reached highly significant differences at 3h and 6h, and its expression trend was consistent with that of the FcRbohD gene (**Figure 4B**). Multiple sequence comparisons showed that the FcMYB3 shared a highly homologous R2-R3 DNA-binding domain at the N-terminus and a highly variable, truncated C-terminal region with other R2R3-MYBs, indicating that FcMYB3 belongs to the R2R3-MYB transcription factor family (**Figure 4C**).

To gain insight into the function of *FcMYB3*, transient knock-down (VIGS) and over-expressing (OE) *F. carica* callus were produced. RT-qPCR analyses of *FcMYB3* expression showed a reduction of 4.16 times relative to the empty control pTRV2::00 vector in the VIGS callus, while in the OE callus, a 3.68 times increase relative to the control empty pCAMBIA1300::00 vector was achieved (**Figure 4D**).

The effect of altered *FcMYB3* expression on *FcRbohD* expression in the callus lines was then tested. In the VIGS callus relative to the empty pTRV2::00 vector, *FcRbohD* expression was decreased by 2.13 times. Conversely, in the OE callus, the expression level of *FcRbohD* relative to the empty pCAMBIA1300::00 vector was increased by 2.36 times (**Figure 4E**).

To investigate the effect of altered *FcMYB3* on callus H_2_O_2_ levels, the transiently transformed callus lines were transferred to the dark for 3 days. The results are shown in **Figure 4D**. The transient knock-down of *FcMYB3* resulted in a reduced level of H_2_O_2_ relative to the callus transformed with the empty control pTRV2::00 vector, while the OE callus showed an increased content of increased H_2_O_2_ relative to the control callus transformed with the control vector, pCAMBIA1300::00 (**Figure 4F**).

### 3.8 FcMYB3 interacts with the *FcRbohD* promoter

**Figure 5:**
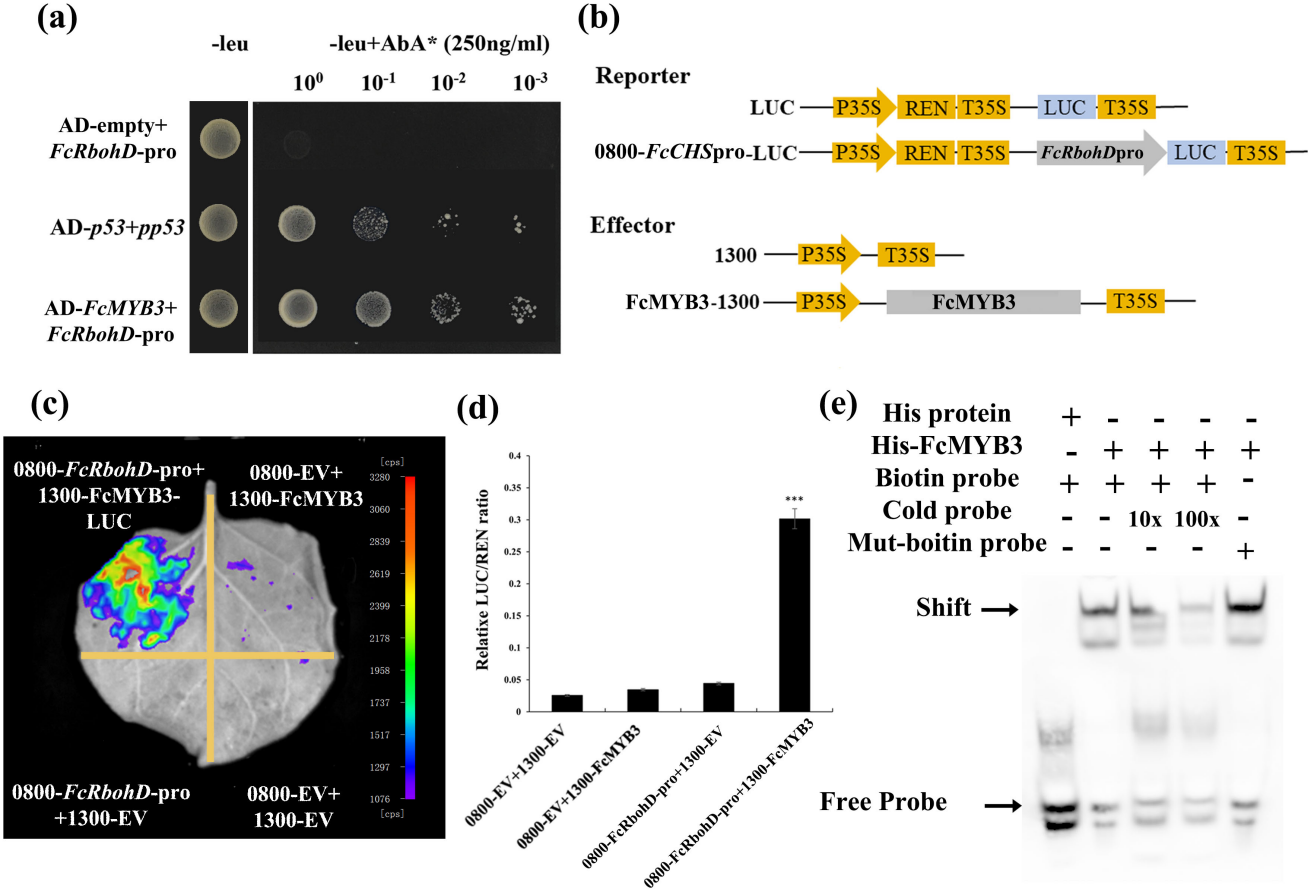
FcMYB3 interacts with Pro-FcRbohD. **(A)** The yeast one -hybrid assay revealing the interaction between FcMYB3 and FcRbohD-pro. **(B)** The constructs used in the dual-luciferase reporter assay. **(C)** Detection of the LUC signal in tobacco leaves. **(D)** The effects of FcMYB3 on the FcRbohD promoter activity, as demonstrated by the luciferase reporter assay. The values are means ± SD of six independent biological replicates. The statistical significance was determined using Student’s t-test (*P < 0.05; ***P < 0.001). **(E)** EMSA analysis indicating that FcMYB3 binds to the TAACTG motifs in the FcRbohD promoters. The hot probe was a biotin-labeled promoter fragment containing the TAACTG motif, whereas the cold probe was an unlabeled competitive probe (250-fold probe concentration). Mutant probes were unlabeled hot probes containing two nucleotide mutations (TTTCTG).

A yeast one-hybrid (Y1H) assay demonstrated that FcMYB3 was capable of binding to the promoter region of *FcRbohD* (**Figure 5A**). To confirm that FcMYB3 is capable of promoting *FcRbohD* expression *in planta*, a LUC assay was performed with the transient co-expression of 35S-*FcMYB3* and the FcRbohD-LUC in tobacco leaves (**Figure 5B**). Relative to control levels, the luminescence intensity resulting from the co-expression was significantly higher (ca. 4 x) (**Figure 5C**). These results indicate that FcMYB3 can activate the transcription of FcRbohD by binding directly to its promoter (**Figure 5D**).

The FcRbohD promoter was seen to contain a MYB-specific binding motif (CAACAG; **Figure 3G**). An electrophoretic mobility shift assay (EMSA) result confirmed that FcMYB3 can bind to the *FcRbohD* promoter fragment containing this motif (**Figure 5E**). Increases in the concentration of the unlabeled competitor probe gradually decreased the observed binding. Alterations of the CAACAG motif to CTTCAG eliminated the binding, even in the absence of the competitor probe, which strongly suggests that FcMYB3 binds to the *FcRbohD* promoter at this motif.

## 4 Discussion

### 4.1 The mechanisms to repair DNA damage in radiation

Maintaining the stability of genetic material is important for the genetic and developmental processes of organisms. For example, UV light could cause up to about 100,000 DNA damages per cell in a single day (Hoeijmakers, 2009). Ionizing radiation can cause both single-strand breaks (SSBs) or double-strand breaks (DSBs) in the DNA double helix (Lord and Ashworth, 2012). During the long evolutionary process, organisms have evolved a series of complex and rigorous mechanisms to repair DNA damage, including base excision repair (BER), nucleotide excision repair (NER), homologous recombination (HR) and non-homologous end joining (NHEJ) (Jasin and Rothstein, 2013; Marteijn et al., 2014). Deletions and insertions involve the removal or addition of segments of DNA respectively. These segments can range from individual base-pairs to several thousand.

If DNA double-strand breaks are not repaired cells may undergo one of several fates: apoptosis, cellular senescence, mutation, or genomic instability. Although senescent cells do not replicate, they may avoid clearance to persist in tissues while continuing to induce stress in neighboring cells. Radiation-induced cellular senescence is an important mediator of tissue dysfunction promoting damage reduction in ROS via the glutathione pathway. During radiation, the production of ROS leads to DNA, protein, and lipid membrane damage. The flavonoid quercetin acts as Antioxidants Peroxisomal Proliferator-Activated Receptor Agonists (Huey et al., 2006). Erroneous repair of DSBs can lead to gross genomic rearrangement. DSB repairs utilize at least two distinct pathways: homologous recombination (HR) and nonhomologous end-joining (NHEJ). HR is a critical pathway for the accurate repair of DSBs and maintenance of genomic stability. NHEJ repairs show a decreased fidelity compared to HR. Significant capacity that plants have for DNA repair the plant life cycle will encounter various biotic or abiotic factors during the process of growth and development.

### 4.2 DNA damage response in the fig with 60Co-γ radiation

The DNA damage response (DDR) is an important mechanism evolved by organisms to maintain the stability of genetic material (Lempiainen and Halazonetis, 2009). What’s more, DDR is a signal transduction pathway that detects DNA damage and transduces the signal to downstream regulators to activate related pathways to arrest the cell cycle and repair DNA damage. In this study, most of the genes involved in double-strand break (DSB) repair were significantly up-regulated at 6 h after radiation and are responsible for homologous recombination (HR) repair, mitosis, meiosis and DSB repair. Of these, two DNA helicase DEGs with FPKM ≥ 20 showed early and significant up-regulation at 3–6 h. However, these increases were not sustained and expression levels were reduced from 12–48 h. Nineteen genes were identified as DNA repair protein, and most of them were down-regulated. Two genes were further screened with FPKM ≥ 20. Of these, c43096_g1 showed down-regulation of (FC) 0.80–0.95, 3–48 h after irradiation. The FPKM of c65988_g1 presented the highest value (90.2) in the control and showed a gradual downward trend over time. Forty-four genes were annotated as DNA ligase, and most of them were seen to be up-regulated (31). Two of these with a FPKM ≥ 20 (c46089_g7 and c41717_g4) showed fold changes of 6.26 and 2.48 at 3 and 6 h, respectively. Following their peak expression levels, both genes showed a relative decrease in expression (**Supplementary Figure S2**).

### 4.3 ATM and ATR plays key roles in DNA damage responses

In animal cells, ATM (ataxia-telangiectasia mutated) and ATR (ATM- and Rad3-related) are members of the PIKKs (phosphatidylinositol-3- Kinase-like kinases) family with key roles in regulation of double-stranded and single-stranded DNA damage responses including BER, HR and NHEJ (Lempiainen and Halazonetis, 2009). There are connections between the ATM and ATR signaling pathways and many downstream genes responsible for DNA damage repair are regulated by both. Studies had shown that ATM and ATR regulate the expression of the poly (ADP-ribose) polymerases PARP1 and PARP2. In ATM, ATR double mutants, the induction of PARP1 and PARP2 expression in response to ionizing radiation was significantly inhibited (Culligan et al., 2006). PARG (poly {ADP-ribose} glycohydrolase) is an enzyme that catalyzes the reverse reaction of poly ADP ribosylation modification. PARPs and PARG1 play important roles in the survival of *Arabidopsis* cells. The expression of PARG1 is regulated by ATM and ATR, but in turn can also affect the expression of ATM and ATR (Zhang et al., 2015a). In our study, we found that ionizing radiation caused no significant changes in ATR expression, but an ATM gene (c40272_g1) was found to be upregulated and a PARG1 gene (c37604_g1) to be downregulated 6 h after irradiation. It is therefore possible that the upregulation of the ATM gene observed in irradiated fig buds might negatively regulate the expression of c37604_g1_PARG1 or vice-versa. It is known that, DNA ligase Rad51 responds to irradiation in an ATM-dependent manner (Benson et al., 1998; Bleuyard et al., 2005). Three fig RAD51 genes were identified in this study (c43137_g2, c44721_g1, c40125_g1) and found to be significantly upregulated at 3 and 6 h post-irradiation, one of which, (c43137_g2), maintained higher expression levels relative to the control until 48 h.

### 4.4 ROS signal and plant resistance

The biological effect of γ radiation is based on its interaction with atoms or molecules in the cell, particularly water, to produce ROS such as superoxide, peroxide, singlet oxygen, and hydroxyl radicals, which are natural byproducts of aerobic metabolism (Beyaz et al., 2016; Beyaz, 2019). ROS are products of cell metabolism and can be produced in almost all regions of the organism. There are two common mechanisms of ROS defense: enzymatic and the non-enzymatic antioxidant defense system. Under normal growth conditions, intracellular ROS are maintained at in homeostasis, while conditions of stress and specific developmental signals can cause ROS levels to increase transiently or continuously (Dat et al., 2000). Recent studies have revealed some key proteins involved in ROS signal transduction in the model plant, Arabidopsis (Qi et al., 2018). Although the mechanism of plant ROS perception has not yet been identified, current research suggests that plant cells may sense ROS signals through unknown ROS receptor proteins; redox-sensitive transcription factors such as NPR1, HSF or *via* ROS inhibition of protein phosphatase activity (Neill et al., 2002; Apel and Hirt, 2004). In our study, there were 144, 191, 122, 27 and 44 genes annotated as serine/threonine protein kinases at 3, 6, 12, 24 and 48 h, respectively (**Supplementary Table S3**).

ROS can also promote changes in the expression of different transcription factors, including the WRKY, HSF, GRAS and MYB family members (Desikan et al., 2001; Vandenabeele et al., 2003). A large number of transcription factors regulation were found to be differentially regulated in response to ionizing radiation in our study, with members of the WRKY and MYB families being the most affected (**Table 3**).

It is known that programmed cell death could be triggered by different types of ROS and their effects on macromolecules, including lipid peroxidation (Montillet et al., 2005). ROS accumulation and signaling can lead to an increased stress resistance in plants, probably through the ROS activation of plant defense systems including various kinases, transcription factors, other signaling molecules, antioxidant enzymes, dehydrin, low temperature-inducible proteins, heat shock proteins and disease-associated proteins (Vranova et al., 2002; Moon et al., 2003). NADPH oxidase (NOX) is a key enzyme in the redox signal *in vivo* and a major source of ROS in organisms (Foreman et al., 2003; Turkan et al., 2018). In our study, the differential regulation of 3 NOX genes was significant at 3, 6 and 12 h post-irradiation. A large number of other ROS-related genes was also observed 6 h after the radiation treatment. The non-enzymatic antioxidant defense components such as glutathione, phenylpropanoids, flavonoids, contribute substantially to the regulation of ROS levels. In this study, key genes of their biosynthetic pathways were up-upregulated in response to fig irradiation treatments.

ROS signals are primarily generated by the RBOHD, which is regulated by various types to maintain appropriate dynamics of ROS burst (Fichman et al., 2021). In this study, Both FcRbohD and MYB3’s expression level responded to 60Co-γ radiation, with *FcMYB3* responding earlier than *FcRbohD* (**Figures 2C**, **4A**). Additionally, the interaction between FcMYB3 and the *FcRbohD* promoter supports the hypothesis that MYB3 acts upstream of FcRbohD in the stress response pathway. Knockdown and overexpression of *FcMYB3* in fig callus tissue indicate that FcMYB3 is a positive regulator of ROS accumulation in response to γ-ray radiation stress (**Figure 4F**). The FcMYB3 protein binds to the MYB-specific binding motif (CAACAG) in the *FcRbohD* promoter and positively activates its promoter activity, further demonstrating that the FcMYB3-*FcRbohD* module is involved in the regulation of ROS accumulation. Recent studies have shown that, in addition to direct regulation by transcription factors, RBOHD is modulated by various types of modifications such as phosphorylation, ubiquitination, calcium binding, S-nitrosylation, and persulfidation to maintain appropriate dynamics of ROS bursts (Lee et al., 2020; Qi et al., 2024). The detailed mechanisms by which RBOHD protein activity influences ROS bursts require further investigation.

## Data Availability

The datasets presented in this study can be found in online repositories. The names of the repository/repositories and accession number(s) can be found in the article/**Supplementary Material**.
