# Supplementary material for: The transcription factor FcMYB3 responds to 60Co γ-ray irradiation of axillary buds in Ficus carica L. by activating the expression of the NADPH oxidase, FcRbohD
Source: Front Plant Sci. 2024 Nov 26;15:1476126. doi: 10.3389/fpls.2024.1476126 (PMC11628289; doi:10.3389/fpls.2024.1476126)
Supplement: Supplementary file 1 [file DataSheet1.pdf]

Supplementary materials

Table S1. Primer sequences of genes used for verification of digital gene-expression results by qRT-PCR.

Table S2. Summary of the sequencing assembly.

Table S3. The FPKM of Veen differentially expressed genes (DEGs) in ‘Green peel’ fig dormant branch by ^{60}Co γ-ray radiation mutagenesis.

Table S4. Expression profiles of transposon-related DEGs in Green peel fig dormant branch by ^{60}Co γ-ray radiation mutagenesis.

Figure S1. GO and KEGG enrichment analysis.

Figure S2. qRT-PCR validation. Twenty DEGs were randomly selected to validate the RNA-Seq results. Correlation of fold changes between RNA-Seq and qRT-PCR was analyzed. Three biological replicates were used for the qRT-PCRs.

Figure S3. The full uncropped Gels and Blots LUC image

Figure S4. The full uncropped Gels and Blots EMSA image

Table S1. Primer sequences of genes used for verification of digital gene-expression results by qRT-PCR.

Seq ID	Forward Primer (5'-3')	Reverse Primer (5'-3')
Actin (Reference gene)	GCCATTCAAGCCGTGCTTT	TGGGAACAGTGTGGCTGACA
c46667_g1	AGCCCTACTCTTCCCCTAGG	CCGCATTGTGTAAGGGTTCC
c43190_g1	GAGGCCCCAGTGAAGTTAGA	GCAGCTTATCCCCTTTGTGG
c66287_g1	CCCGACTCTTCCTTCTCTCC	CCGTACCATCATCAAGCACG
c31330_g1	TGGTGGACGTGGTGGATTTA	TTCTTGCTGACAGATCCGGT
c35817_g1	CCGTTGACTGGGGAAAAGTG	TGCAGGAAATTGGGTGAAGC
c65898_g1	TGACGCACCATGTTCTTCAC	GTCGCTTGATGGTGGTTGTT
c11402_g1	CGTCTTGGCTTGAATCCTCC	AGAACGAGGCTGAACACTGT
c23209_g1	CTCCTTTCTTCTTTCCCGCG	ATTCCCTCCTCGCTTTCTCC
c43096_g1	CTTGGTGGGCTGACAATCAC	TCCTCACTTGGTTTCCCTCC
c65988_g1	GGAGGAGGAGGTTCAGTTCC	TAGCGTGGTACCAAGGGATG
c18002_g1	TGGTAAAGTGGGAGGGTGTC	GTAGCGAGTGAGAGAGGGTC
c46726_g1	CGAATCGAACCCTTGCCAAT	TGCCTGTGTCGTTGAAGTTG
c72713_g1	GAAACCCTCTTCCGGACACT	TTCGATCATGTTCCGCCTCT
c40706_g1	TTTACCCCACCCAAGATCCC	TCGAAGAAGAGCGTCTGGTT
c45114_g1	CGTCGGCGTATTCAAGGATG	GCTCTAAGGATTGTGTGGGC
c26575_g1	CTGGAAGTGTGCAAGAGGTG	GCAGATCAAGTGGCACCAAC
c15719_g1	AATCTCCAAAACAGGCCTGC	TAGCTCCGGTGAAAGTGTCA
c43241_g1	GCCTTGAAGTGATATCGGCC	GCCCAAGTTCGAGAAGGTTC
c44569_g1	TGTATTCCTCCGCGAACAGA	ACCTTTCCCTGCATTGCTTG
c30313_g1	TGGGGTAAGGTTAGTGCCAG	AAGCGGAAGAATGACATGGC

Table S2. Summary of the sequencing assembly

Sequences	0 h	3 h	6 h	12 h	24 h	48 h
**Before trimming**						
Total nucleotides (bp)	8,683,200,489	8,710,373,996	9,165,865,496	8,201,280,969	8,111,456,505	7,922,294,728
Number of raw reads	57,504,639	57,684,596	60,701,096	54,313,119	53,718,255	52,465,528
Q20 percentage (%)	97.77	97.81	97.91	97.80	97.76	97.80
Q30 percentage (%)	94.18	94.19	94.43	94.25	94.19	94.16
**After trimming**						
Total nucleotides (bp)	8,497,633,883	8,528,250,511	8,976,775,520	8,032,335,382	7,929,508,542	7,768,855,761
Number of raw reads	56,969,270	57,155,624	60,147,837	53,802,630	53,141,874	52,012,303
Q20 percentage (%)	98.45	98.45	98.52	98.46	98.47	98.43
Q30 percentage (%)	95.05	95.02	95.22	95.09	95.11	94.96
**Mapping ratio**						
Total nucleotides (nt) of transcripts (bp)	28,484,635	28,577,812	30,073,919	26,901,315	26,570,937	26,006,152
Mapped nucleotides (nt) of transcripts (bp)	23,510,535	23,709,167	24,952,247	22,075,327	21,755,496	21,270,473

Mapped rate (%)	82.50	82.94	82.96	82.06	81.88	81.81

Table S3. The FPKM of Veen differentially expressed genes (DEGs) in Green peel fig dormant branch by ^{60}Co γ-ray radiation mutagenesis

Gene_ID	FPKM					
	GR_CK	GR_3	GR_6	GR _12	GR_24	GR_48
**Disease resistance (95)**						
TRINITY_DN30359_c0_g1	0	0.12	0.395	0.08	0.08	0.13
TRINITY_DN33257_c0_g1	0.37	0.885	0.655	1.195	0.725	1.19
TRINITY_DN37121_c0_g1	0	0.095	0.11	0.69	0.12	0
TRINITY_DN43358_c0_g1	0	2.085	1.75	2.165	0.465	0.17
TRINITY_DN43703_c0_g1	3.18	6.105	6.06	4.755	3.585	5.6
TRINITY_DN44464_c0_g1	65.03	85.555	90.395	51.04	45.045	52.41
TRINITY_DN44568_c0_g1	4.805	13.44	16.205	21.005	13.86	21.975
TRINITY_DN44810_c4_g1	27.76	30.49	19.01	14.395	20.755	18.885
TRINITY_DN45033_c0_g1	59.78	423.825	240.425	169.805	90.835	88.465
TRINITY_DN45048_c2_g1	35.665	16.15	12.98	29.62	46.61	48.825
TRINITY_DN45104_c1_g1	10.115	70.67	84.025	79.685	26.15	9.215
TRINITY_DN45181_c0_g1	24.76	11.18	19.055	21	24.395	43.015
TRINITY_DN45283_c2_g1	5.88	5.24	9.995	7.815	5.78	10.635
TRINITY_DN45349_c1_g1	2.13	5.47	12.67	5.725	6.425	8.775
TRINITY_DN45468_c0_g1	0.275	1.42	1.965	11.92	3.525	1.77
TRINITY_DN45514_c1_g1	66.88	28.905	21.125	46.89	49.725	54.45
TRINITY_DN45628_c1_g3	4.735	23.585	26.805	5.925	4.605	7.32
TRINITY_DN45791_c3_g7	0.15	2.595	2.98	0.6	0.37	0.455
TRINITY_DN45836_c2_g1	9.085	12.66	16.23	8.96	11.525	14
TRINITY_DN45942_c3_g1	2.015	3.535	3.44	2.615	1.73	1.235
TRINITY_DN46009_c1_g3	25.29	46.065	48.48	41.94	34.475	25.065

TRINITY_DN46009_c1_g5	9.46	5.02	4.005	3.725	5.89	6.57
TRINITY_DN46218_c1_g3	0.185	1.085	0.87	0.86	0.355	0.36
TRINITY_DN46254_c2_g1	7.17	27.72	18.48	12.9	8.46	8.44
TRINITY_DN46411_c0_g3	0.155	0.43	0.355	0.595	0.42	0.905
TRINITY_DN46427_c0_g4	0.04	0.825	0.98	0.245	0.225	0.12
TRINITY_DN46501_c0_g1	0.21	0.56	1.3	0.43	0.415	0.36
TRINITY_DN46501_c1_g4	3.655	6.725	8.645	8.68	6.42	4.82
TRINITY_DN46552_c2_g1	0.6	1.22	4.15	2.035	1.495	2.76
TRINITY_DN46609_c2_g1	48.345	94.835	109.2	95.41	67.06	61.435
TRINITY_DN46630_c0_g1	3.775	7.79	12.245	5.765	5.725	3.005
TRINITY_DN46795_c2_g2	26.67	27.97	47.525	43.925	28.76	31.145
TRINITY_DN46871_c1_g1	2.785	4.705	6.9	8.795	4.375	2.575
TRINITY_DN46916_c1_g3	20.285	25.73	32.14	36.435	23.565	17.775
TRINITY_DN46929_c2_g3	1.485	0.58	0.475	0.69	0.665	0.83
TRINITY_DN46986_c1_g3	0.12	1.615	1.47	0.605	0.515	0.37
TRINITY_DN46986_c1_g4	0.28	0.42	0.92	0.57	0.37	0.245
TRINITY_DN46994_c0_g4	2.235	4.21	4.44	3.915	2.935	3.18
TRINITY_DN46994_c0_g8	0.1	0.285	0.275	0.78	0.365	0.21
TRINITY_DN47102_c6_g1	12.06	30.895	27.305	22.885	16.22	14.235
TRINITY_DN47189_c1_g2	1.055	0.82	0.57	2.28	2.035	3.3
TRINITY_DN47197_c0_g1	4.685	29.69	20.865	8.825	7.455	7.085
TRINITY_DN47288_c5_g3	3.555	7.265	9.35	4.505	3.18	2.995
TRINITY_DN47347_c3_g2	2.275	5.525	6.18	4.805	3.855	3.265
TRINITY_DN47397_c3_g2	0.655	1.11	1.97	1.875	1.01	1.27
TRINITY_DN47430_c2_g1	62.94	76.99	83	161.805	84.9	47.025
TRINITY_DN47502_c6_g1	5.925	68.52	109.68	168.77	46.06	17.98
TRINITY_DN47720_c3_g2	0.205	0.98	1.065	0.575	0.4	0.41
TRINITY_DN47793_c5_g2	13.16	26.46	29.53	20.165	15.05	16.195

TRINITY_DN47833_c0_g1	1.42	2.255	6.33	4.455	2.98	2.81
TRINITY_DN47850_c1_g1	7.535	12.705	15.135	11.315	9.415	14.615
TRINITY_DN47993_c0_g2	0.41	2.475	2.42	2.05	1.06	0.965
TRINITY_DN47993_c0_g6	0	0.33	0.625	0.785	0.09	0.13
TRINITY_DN48046_c3_g1	0.065	0.755	0.29	0.35	0.09	0.065
TRINITY_DN48073_c3_g2	2.125	3.05	4.785	5.835	3.025	3.03
TRINITY_DN48102_c0_g5	1.595	3.66	6.56	5.005	3.095	4.16
TRINITY_DN48202_c4_g5	2.78	5.495	4.76	7.37	5.14	7.775
TRINITY_DN48646_c3_g2	32.785	26.285	27.12	45.32	51.46	56.785
TRINITY_DN48784_c5_g1	0.565	1.245	1.035	0.94	1.275	2.385
TRINITY_DN48869_c2_g1	1.82	5.225	8.575	7.735	4.315	4.715
TRINITY_DN48881_c1_g2	1432.855	527.395	291.69	297.55	366.01	1109.57
TRINITY_DN48934_c0_g4	0.57	0.835	1.37	0.925	2.275	3.17
TRINITY_DN49019_c4_g1	3.01	6.695	8.195	4.515	3.285	3
TRINITY_DN49021_c0_g1	7.46	16.08	14.18	7.155	6.94	7.71
TRINITY_DN49043_c7_g1	40.28	19.955	40.73	47.63	38.61	37.495
TRINITY_DN49066_c0_g1	1.085	6.045	5.29	2.27	2.005	1.165
TRINITY_DN49076_c0_g1	5.165	0.895	0.595	0.63	1.85	4.89
TRINITY_DN49076_c0_g2	6.615	13.285	6.785	9.93	18.99	12.28
TRINITY_DN49136_c1_g1	5.48	16.615	12.075	7.745	6.315	5.49
TRINITY_DN49174_c3_g1	5.805	1.57	0.765	4.65	3.98	5.71
TRINITY_DN49209_c0_g1	4.975	12.735	15.705	11.075	10.155	20.765
TRINITY_DN49209_c0_g3	1.7	2.55	3.295	2.37	2.13	2.43
TRINITY_DN49270_c1_g2	0.03	0.725	0.89	0.955	0.37	0.265
TRINITY_DN49308_c4_g5	41.78	26.415	15.215	39.935	23.635	33.11
TRINITY_DN49391_c4_g5	34.885	63.35	103.425	73.945	56.37	59.585
TRINITY_DN49446_c2_g2	0.255	0.81	1.39	2.65	0.725	0.235
TRINITY_DN49599_c1_g3	11.695	3.245	2.44	2.575	3.75	10.815

TRINITY_DN49610_c1_g1	38.705	52.475	61.04	63.685	40.805	30.295
TRINITY_DN49726_c0_g1	16.145	26.225	31.99	32.535	25.44	29.7
TRINITY_DN49744_c0_g3	23.47	80.48	84.84	46.135	40.41	28.635
TRINITY_DN49744_c0_g4	7.94	16.425	14.695	11.985	11.75	8.355
TRINITY_DN49756_c1_g2	1.895	3.055	4.665	4.315	2.44	2.13
TRINITY_DN49780_c1_g1	0.68	3.825	3.755	1.815	0.64	0.495
TRINITY_DN49827_c1_g1	4.085	6.93	3.115	4.375	3.795	4.815
TRINITY_DN49839_c3_g1	1.165	11.82	9.6	3.745	2.01	2.965
TRINITY_DN49839_c3_g6	1.61	5.055	5.62	3.71	2.23	3.595
TRINITY_DN49851_c3_g1	5.075	9.09	9.545	5.99	5.76	5.7
TRINITY_DN49883_c2_g1	7.37	10.34	11.24	5.725	7.4	8.575
TRINITY_DN49924_c1_g1	11.5	21.885	27.075	27.825	19.3	33.06
TRINITY_DN49933_c4_g5	0.465	1.965	2.59	2.72	1.485	1.935
TRINITY_DN49950_c9_g3	3.06	8.215	10.38	8.205	5.075	6.175
TRINITY_DN49956_c9_g2	13.02	25.7	29.875	24.88	20.075	13.63
TRINITY_DN49956_c9_g3	12.63	10.25	19.57	15.685	7.41	8.57
TRINITY_DN49959_c9_g1	4.73	1.32	1.205	4.105	4.44	8.32
TRINITY_DN49959_c9_g2	8.175	5.035	4.325	10.72	12.005	18.585
**Triacylglycerol lipase (7)**						
TRINITY_DN43008_c0_g1	8.94	5.395	6.79	28.635	10.855	10.41
TRINITY_DN45326_c0_g1	7.425	10.175	7.255	13.665	9.27	9.89
TRINITY_DN45481_c2_g1	638.925	50.225	108.725	101.6	435.9	137.665
TRINITY_DN46081_c5_g2	2.97	25.275	35.68	95.555	44.42	19.155
TRINITY_DN46500_c1_g1	8.32	22.48	14.46	13.36	8.46	7.715
TRINITY_DN48040_c2_g1	27.565	9.01	6.675	14.82	28.1	30.555
TRINITY_DN49310_c2_g1	12.545	11.75	26.32	14.415	11.015	11.51
**Lipid transfer protein (LTP) family protein (19)**						
TRINITY_DN16314_c0_g1	210.39	74.83	47.795	94.59	368.775	244.565

TRINITY_DN40425_c0_g1	12.635	14.21	5.495	3.32	6.63	2.095
TRINITY_DN41345_c0_g1	2.05	15.845	18.92	23.96	1.43	0.75
TRINITY_DN44246_c4_g9	34.925	13.89	9.74	71.995	196.01	553.61
TRINITY_DN44565_c5_g1	20.115	60.495	47.67	48.865	28.275	21.505
TRINITY_DN45206_c3_g3	41.78	6.71	5.04	9.38	30.525	49.235
TRINITY_DN46430_c2_g12	62.595	2.41	3.7	16.44	101.395	152.41
TRINITY_DN46835_c7_g4	5.735	4.91	3.54	8.125	12.13	24.475
TRINITY_DN47069_c1_g1	1235.175	549.085	484.48	1231.21	962.26	567.965
TRINITY_DN47069_c1_g3	3.805	0.665	0.475	0.775	1.42	1.03
TRINITY_DN47069_c2_g1	10688.55	5617.69	4035.695	6390.94	2843.48	571.495
TRINITY_DN47069_c3_g2	3054.015	1375.595	1369.59	2930.645	2307.085	1555.385
TRINITY_DN47113_c7_g6	29.36	7.425	5.01	27.565	37.98	37.755
TRINITY_DN47138_c1_g8	96.605	68.235	47.52	73.875	79.005	30.695
TRINITY_DN47402_c2_g5	98.23	31.44	28.37	28.485	54.88	63.59
TRINITY_DN47727_c5_g3	13.975	7.22	1.995	3.04	2.325	0.23
TRINITY_DN49192_c5_g1	4.055	1.14	0.57	0.15	0.74	1.065
TRINITY_DN49593_c5_g1	415.375	224.275	180.645	162.685	258.67	188.54
TRINITY_DN55153_c0_g1	9.54	14.88	7.35	5.365	11.01	2.34
**Germin-like protein subfamily 1 member (3)**						
TRINITY_DN43118_c0_g1	6.98	31.155	24.405	11.875	8.465	6.79
TRINITY_DN47085_c2_g3	9.395	0.78	0.33	0.605	0.76	0.395
TRINITY_DN47852_c3_g1	6.59	15.21	10.64	33.13	16.42	14.4
**E3 ubiquitin-protein ligase (89)**						
TRINITY_DN34190_c0_g1	0.645	15.675	24.185	15.365	3.15	0.755
TRINITY_DN37461_c0_g1	0.035	1.34	2.225	0.735	0.215	0.34
TRINITY_DN42182_c0_g1	1.755	5.325	5.32	2.91	3.74	3.45
TRINITY_DN42226_c0_g1	54.78	17.065	10.105	9.385	23.755	27.64
TRINITY_DN42761_c0_g1	2.63	10.405	6.39	4.03	2.155	2.19

TRINITY_DN43903_c1_g1	0.015	1.795	0.385	0.08	0.285	0.34
TRINITY_DN43922_c0_g1	15.09	34.03	30.565	22.09	16.97	17.155
TRINITY_DN44146_c3_g15	232.39	287.82	519.325	582.065	490.965	410.65
TRINITY_DN44161_c0_g1	0.23	2.76	1.285	0.675	0.24	0.09
TRINITY_DN44161_c0_g2	0.03	0.795	0.385	0.19	0.03	0.08
TRINITY_DN44161_c0_g4	0	1.01	0.73	0.18	0.06	0.04
TRINITY_DN44592_c5_g2	7.145	89.655	119.8	83.605	50.485	33.385
TRINITY_DN44601_c0_g1	26.815	22.48	12.035	19.025	17.865	11.94
TRINITY_DN44651_c3_g2	0.39	1.9	1.025	2.25	1.935	1.135
TRINITY_DN44669_c1_g1	0.23	0.87	1.715	0.84	0.775	0.895
TRINITY_DN44894_c0_g4	2.49	3.885	6.465	8.535	9.985	13.125
TRINITY_DN44999_c4_g2	111.495	402.775	314.075	214.93	143.14	125.35
TRINITY_DN45054_c1_g1	49.31	29.545	25.125	18.69	22.075	18.065
TRINITY_DN45124_c1_g1	24.895	26.435	42.29	73.64	35.59	24.195
TRINITY_DN45124_c2_g1	19.98	45.155	46.845	34.12	22.525	21.62
TRINITY_DN45153_c2_g1	9.72	6.54	2.115	6.695	8.49	13.735
TRINITY_DN45217_c5_g3	17.52	53.975	47.825	13.725	24.095	80.885
TRINITY_DN45323_c3_g2	64.795	48.08	141.38	251.97	184.82	147.9
TRINITY_DN45324_c1_g8	65.63	78.13	133.16	79.935	73.71	62.34
TRINITY_DN45461_c3_g4	33.715	37.98	59.29	69.305	52.27	56.42
TRINITY_DN45570_c4_g1	6.69	3.285	1.745	3.43	4.9	5.645
TRINITY_DN45650_c0_g3	0.5	3.285	4.865	1.895	1.48	1.71
TRINITY_DN45656_c1_g3	45.365	82.82	90.57	68.57	59.035	62.62
TRINITY_DN45680_c2_g1	0.185	7.05	7.36	4.17	1.1	0.255
TRINITY_DN45733_c0_g6	7.275	2.31	1.905	3.735	5.755	8.78
TRINITY_DN45782_c2_g1	51.91	26.465	14.285	13.155	31.28	33.435
TRINITY_DN45911_c4_g1	38.2	43.6	71.635	84.485	55.72	57.85
TRINITY_DN45932_c3_g7	2.82	1.635	0.83	1.89	1.745	1.645

TRINITY_DN45932_c3_g9	23.295	15.095	9.58	21.165	18.155	16.01
TRINITY_DN46273_c1_g2	1.29	0.61	0	0.385	2.425	3.56
TRINITY_DN46328_c0_g3	0.42	51.115	16.405	4.55	1.19	0.65
TRINITY_DN46349_c3_g1	5.805	11.23	14.235	9.295	6.14	3.78
TRINITY_DN46353_c0_g1	17.22	43.76	63.155	23.565	19.81	25.035
TRINITY_DN46380_c0_g1	4.325	12.16	11.17	10.08	5.765	2.975
TRINITY_DN46393_c2_g4	6.98	4.605	2.725	4.285	6.38	5.89
TRINITY_DN46455_c1_g2	6.77	6.67	2.8	9.205	5.58	3.155
TRINITY_DN46539_c0_g1	0.045	2.38	2.095	0.695	0.025	0.125
TRINITY_DN46550_c0_g1	9.1	7.9	3.26	5.875	6.645	5.695
TRINITY_DN46594_c3_g1	39.56	64.98	72.175	58.09	51.34	50.85
TRINITY_DN46638_c3_g2	6.325	7.035	5.975	9.1	12.295	15.035
TRINITY_DN46651_c1_g1	17.17	14.99	6.94	15.25	12.635	12.54
TRINITY_DN46714_c2_g6	42.64	18.36	29.39	61.295	42.895	47.93
TRINITY_DN46757_c6_g1	198.1	393.51	521.29	294.47	247.43	275.385
TRINITY_DN46757_c6_g2	1.23	8.805	6.37	2.505	1.045	0.77
TRINITY_DN46765_c1_g9	0.425	1.255	1.93	0.595	0.54	0.475
TRINITY_DN46819_c2_g4	18.705	58.315	114.425	116.925	43.46	24.45
TRINITY_DN46906_c2_g1	31.615	140.67	182.15	58.2	39.945	36.68
TRINITY_DN46914_c0_g1	6.03	10.12	23.075	30.29	14.32	15.245
TRINITY_DN47066_c3_g2	18.85	35.03	34.71	27.68	21.125	21.445
TRINITY_DN47093_c2_g1	17.925	5.635	4.905	12.525	13.94	11.31
TRINITY_DN47155_c2_g5	9.72	8.445	12.185	30.17	39.785	71.87
TRINITY_DN47237_c4_g3	0.465	3.805	4.255	2.78	1.68	1.195
TRINITY_DN47305_c1_g1	23.405	71.995	52.72	39.525	31.225	24
TRINITY_DN47322_c1_g2	40.91	118.46	34.045	17.855	14.56	6.725
TRINITY_DN47400_c4_g5	13.285	2.57	1.61	7.785	8	8.225
TRINITY_DN47669_c4_g3	10.05	115.06	56.345	19.43	12.78	13.425

TRINITY_DN47669_c4_g4	3.355	44.235	17.36	8.22	7.275	10.745
TRINITY_DN47692_c5_g1	3.88	14.42	24	11.64	8.295	9.87
TRINITY_DN47696_c0_g1	0.56	1.195	1.66	2.31	0.82	0.4
TRINITY_DN47822_c1_g1	1.535	8.1	4.25	0.995	0.795	1.615
TRINITY_DN47878_c1_g5	5.145	15.9	16.975	18.585	13.11	7.22
TRINITY_DN47918_c4_g1	3.185	7.065	10.365	9.185	4.375	9.11
TRINITY_DN47970_c2_g1	30.495	28.54	42.77	61.475	37.41	25.08
TRINITY_DN47984_c5_g1	85.725	542.01	374.75	190.275	145.48	107.225
TRINITY_DN48084_c0_g2	5.815	2.6	2.24	2.72	3.595	4.305
TRINITY_DN48277_c1_g1	0.585	0.49	1.285	2.82	0.89	1.875
TRINITY_DN48288_c2_g1	38.31	81.77	166.945	71.035	55.405	45.355
TRINITY_DN48288_c2_g8	0.33	1.21	1.42	0.795	0.435	0.555
TRINITY_DN48302_c2_g2	4.47	1.4	0.855	2.175	3	4.345
TRINITY_DN48350_c5_g4	5.705	5.795	2.315	2.59	4.29	3.685
TRINITY_DN48354_c2_g1	2.245	1.31	0.975	0.85	1.315	1.3
TRINITY_DN48553_c2_g2	33.17	70.975	134.93	72.665	36.325	35.59
TRINITY_DN48605_c1_g1	0.06	0.49	0.61	1.865	0.185	0.435
TRINITY_DN48711_c4_g1	0.205	2.635	1.265	0.325	0.42	0.78
TRINITY_DN48711_c4_g4	34.175	104.435	73.26	39.125	30.965	42.47
TRINITY_DN48853_c5_g2	341.88	110.82	77.5	171.28	203.13	92.915
TRINITY_DN48952_c2_g2	6.025	3.38	1.955	2.84	4.62	6.16
TRINITY_DN48959_c0_g3	28.7	13.69	12.83	21.135	25.345	30.33
TRINITY_DN48980_c3_g2	22.525	47.555	41.725	28.02	22.7	21.775
TRINITY_DN49268_c0_g1	149.01	137.86	106.415	108.28	87.495	57.345
TRINITY_DN49268_c0_g2	25.69	13.39	40.165	138.725	142.41	133.43
TRINITY_DN49314_c0_g2	31.205	67.01	42.93	34.435	30.85	28.625
TRINITY_DN49731_c3_g1	10.38	262.595	102.175	35.835	19.44	20.785
TRINITY_DN49757_c3_g1	0.985	2.795	4.635	3.1	3.415	7.83

**Stress-associated protein (4)**						
TRINITY_DN45403_c2_g2	57.845	60.91	136.88	99.6	87.27	81.495
TRINITY_DN45486_c4_g1	415.585	581.47	936.415	945.18	908.195	652.685
TRINITY_DN47002_c4_g1	20.905	24.935	20.995	17.49	15.96	8.775
TRINITY_DN49835_c3_g3	88.265	62.065	140.545	229.79	199.565	140.93

Table S4. Expression profiles of transposon-related DEGs in Green peel fig dormant branch by ^{60}Co γ-ray radiation mutagenesis

Gene_ID	0 h FPKM	Treatment FPKM	Log2 FC	P-value	Regulated	Annotation
**3 h vs. 0 h**						
c28379_g1	0.835	8.675	3.41	3.53E-15	up	Reverse transcriptase
c47199_g1	1.77	7.55	2.11657	6.25E-237	up	Reverse transcriptase
c40385_g1	1.54	0.2	-2.92212	9.58E-20	down	Reverse transcriptase
c47195_g2	0	19.365	6.272579	0.005394	up	Transposon
c31402_g1	1.69	28.09	4.097442	8.02E-54	up	Transposon
c54858_g1	0.4	3.185	3.006394	0.040416	up	Transposon
c23077_g1	0.65	4.525	2.834354	3.6E-06	up	Transposon
c44674_g1	10.545	4.495	-1.20544	8.77E-05	down	Transposon
**6 h vs. 0 h**						
c28379_g1	0.835	5.01	2.84	9.98E-09	up	Reverse transcriptase
c47172_g4	1.38	2.455	1.086393 71174	0.008343	up	Reverse transcriptase
c47199_g1	1.77	5.905	1.987362	5.16E-15	up	Reverse transcriptase
c46416_g1	3.48	1.425	-1.03722	0.036955	down	Reverse transcriptase
c43059_g1	1.46	0.19	-2.63944	0.000795	down	Reverse transcriptase

c40385_g1	1.54	0.1	-3.68663	0.001461	down	Reverse transcriptase
c36014_g1	0.05	0.98	4.570211	0.004391	up	Transposon
c44513_g1	0.065	1.21	4.477125	0.006072	up	Transposon
c31402_g1	1.69	14.16	3.302824	2.39E-30	up	Transposon
c23077_g1	0.65	2.945	2.438099	0.000357	up	Transposon
c30416_g1	1.715	3.715	1.396605	0.004034	up	Transposon
c47172_g3	2.01	3.23	1.121411	0.045791	up	Transposon
c46291_g2	5.795	1.95	-1.32606	0.000803	down	Transposon
c45809_g1	13.75	4.315	-1.55647	3.92E-07	down	Transposon
c46962_g1	5.835	1.48	-1.72479	3.23E-05	down	Transposon
c44674_g1	10.545	2.465	-1.84494	1.27E-07	down	Transposon
**12 h vs. 0 h**						
c40385_g1	1.54	0.235	-2.54066	0.03019	down	Reverse transcriptase
c31402_g1	1.69	4.845	1.720623	1.26E-05	up	Transposon
c30416_g1	1.715	3.935	1.393845	0.005319	up	Transposon
c45809_g1	13.75	5.77	-1.23969	3.98E-05	down	Transposon
c46291_g2	5.795	2.14	-1.24124	0.001271	down	Transposon
c47013_g1	7.79	2.75	-1.2942	1.26E-07	down	Transposon
c44674_g1	10.545	3.355	-1.4646	2.49E-06	down	Transposon
**48 h vs. 0 h**						

c28379_g1	0.835	2.98	1.798191	0.016139	up	Putative transposon Ty5-1 protein YCL075W
c31402_g1	1.69	4.785	1.467722	0.002036	up	Transposon

* P-value ≤ 0.05 and absolute value of $\log_2$FC ratio ≥ 1 (2-fold) as the threshold.

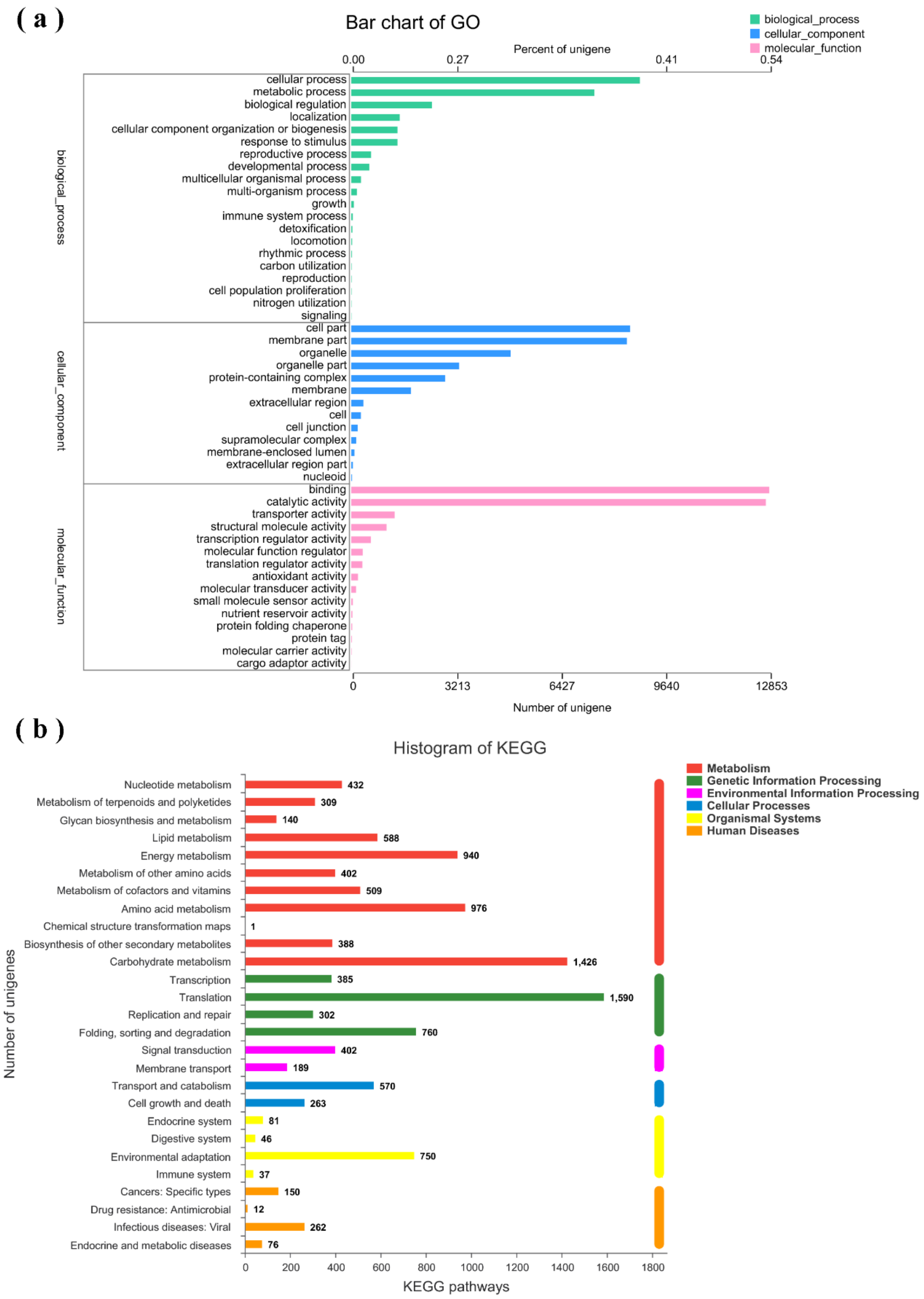

Figure S1. GO and KEGG analysis

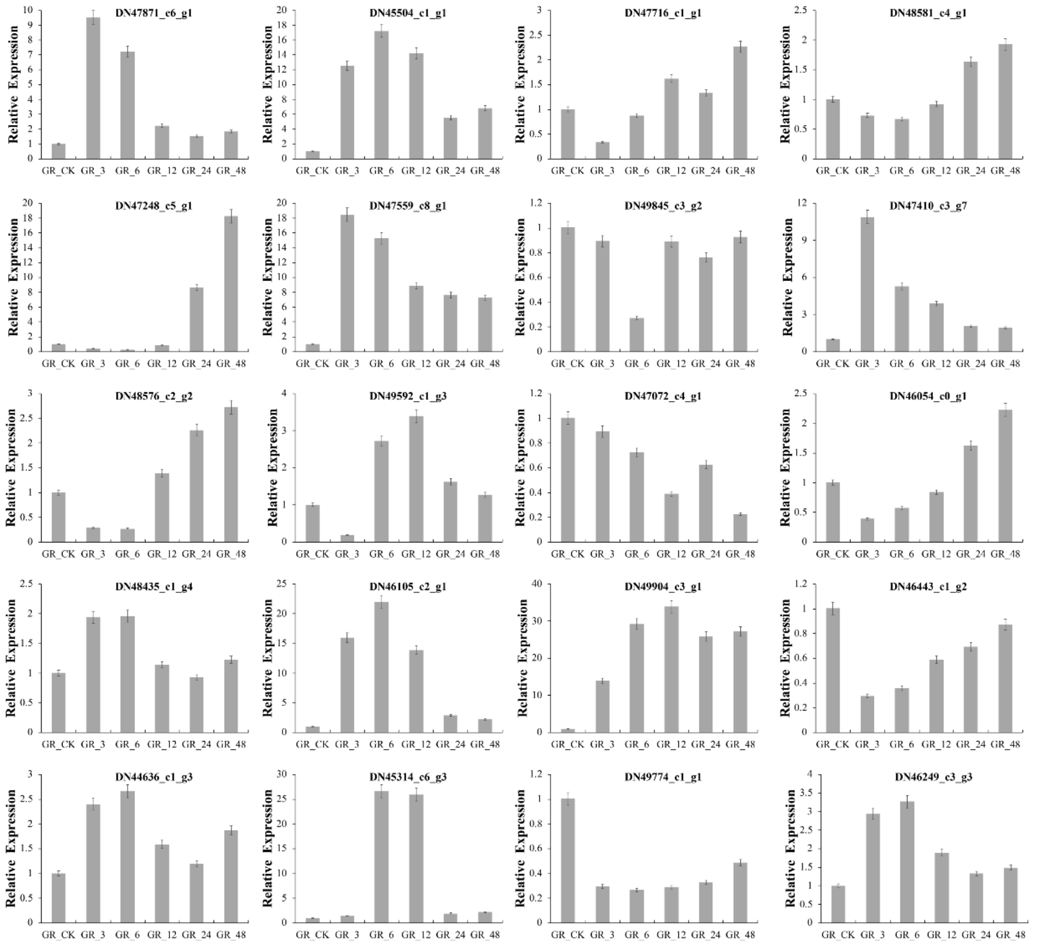

Figure S2. qRT-PCR validation. Twenty DEGs were randomly selected to validate the RNA-Seq results. Correlation of fold changes between RNA-Seq and qRT-PCR was analyzed. Three biological replicates were used for the qRT-PCRs.

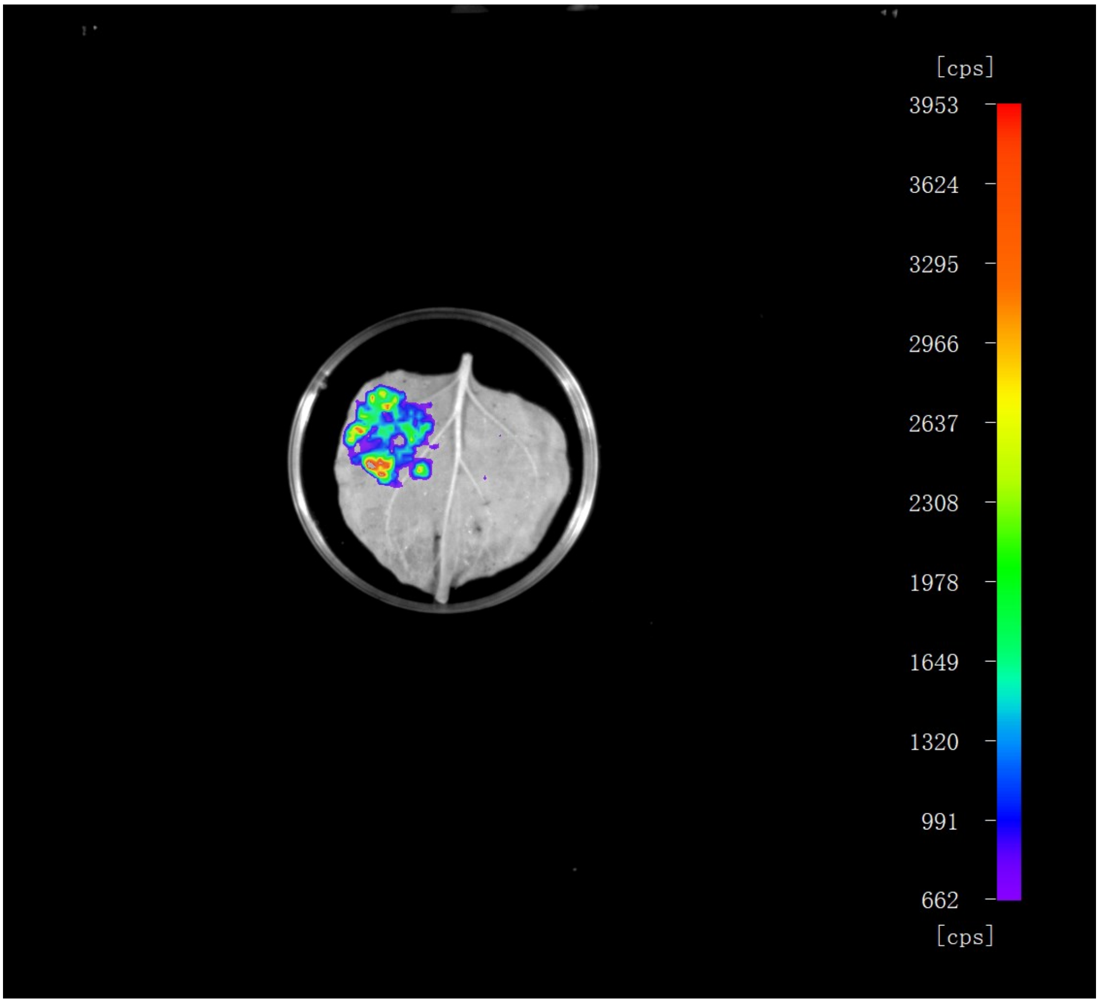

Figure S3. The full uncropped Gels and Blots LUC image

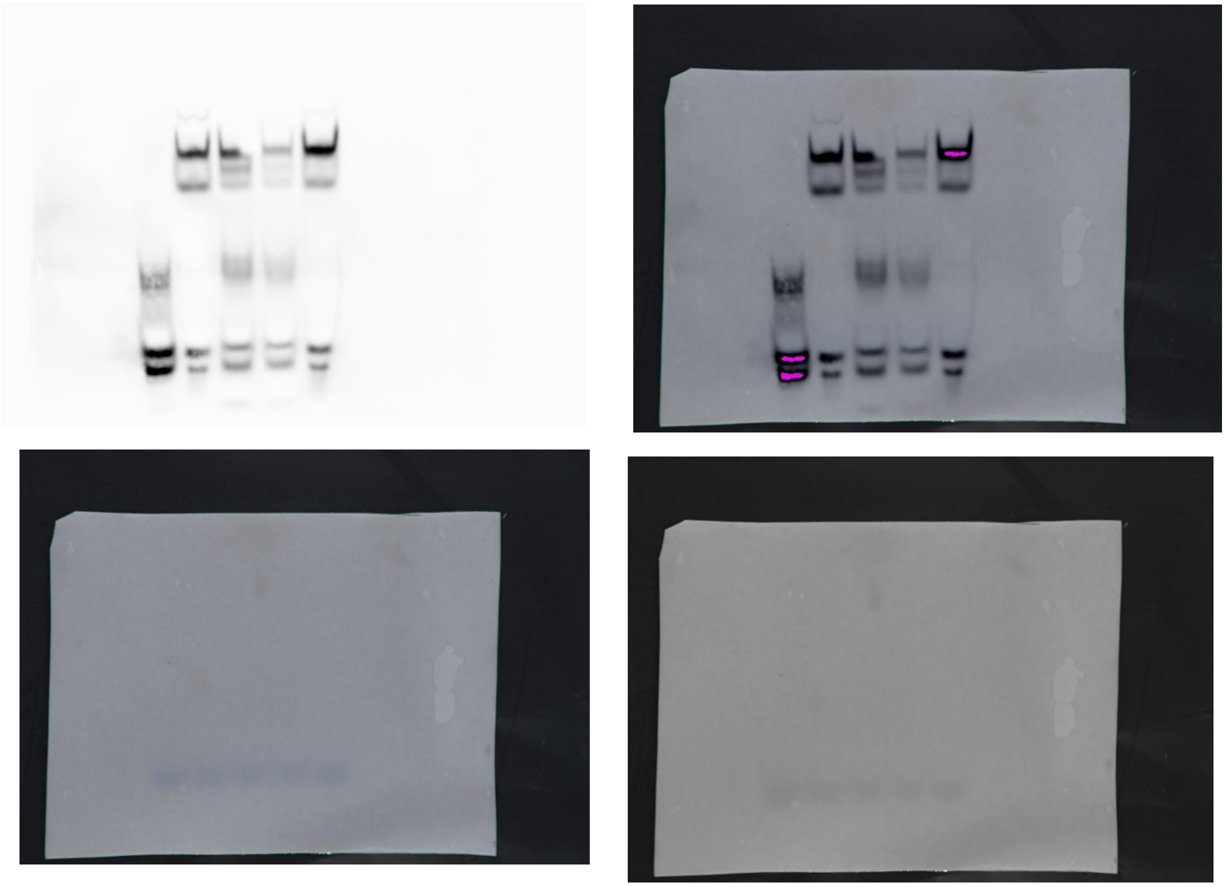

Figure S4. The full uncropped Gels and Blots EMSA image
